# Causal genetic regulation of DNA replication on immune microenvironment in colorectal tumorigenesis: Evidenced by an integrated approach of trans-omics and GWAS

**DOI:** 10.7555/JBR.37.20230081

**Published:** 2023-12-18

**Authors:** Sumeng Wang, Silu Chen, Huiqin Li, Shuai Ben, Tingyu Zhao, Rui Zheng, Meilin Wang, Dongying Gu, Lingxiang Liu

**Affiliations:** 1 Department of Oncology, the First Affiliated Hospital of Nanjing Medical University, Nanjing, Jiangsu 210029, China; 2 Department of Environmental Genomics, Jiangsu Key Laboratory of Cancer Biomarkers, Prevention and Treatment, Collaborative Innovation Center for Cancer Personalized Medicine, Nanjing Medical University, Nanjing, Jiangsu 211166, China; 3 Department of Genetic Toxicology, the Key Laboratory of Modern Toxicology of Ministry of Education, Center for Global Health, School of Public Health, Nanjing Medical University, Nanjing, Jiangsu 211166, China; 4 Department of Biostatistics, Center for Global Health, School of Public Health, Nanjing Medical University, Nanjing, Jiangsu 211166, China; 5 Department of Oncology, Nanjing First Hospital, Nanjing Medical University, Nanjing, Jiangsu 210006, China

**Keywords:** trans-omics, DNA replication, tumor immune microenvironment, causal mediation, colorectal tumorigenesis

## Abstract

The interplay between DNA replication stress and immune microenvironment alterations is known to play a crucial role in colorectal tumorigenesis, but a comprehensive understanding of their association with and relevant biomarkers involved in colorectal tumorigenesis is lacking. To address this gap, we conducted a study aiming to investigate this association and identify relevant biomarkers. We analyzed transcriptomic and proteomic profiles of 904 colorectal tumor tissues and 342 normal tissues to examine pathway enrichment, biological activity, and the immune microenvironment. Additionally, we evaluated genetic effects of single variants and genes on colorectal cancer susceptibility using data from genome-wide association studies (GWASs) involving both East Asian (7062 cases and 195745 controls) and European (24476 cases and 23073 controls) populations. We employed mediation analysis to infer the causal pathway, and applied multiplex immunofluorescence to visualize colocalized biomarkers in colorectal tumors and immune cells. Our findings revealed that both DNA replication activity and the flap structure-specific endonuclease 1 (*FEN1*) gene were significantly enriched in colorectal tumor tissues, compared with normal tissues. Moreover, a genetic variant rs4246215 G>T in *FEN1* was associated with a decreased risk of colorectal cancer (odds ratio = 0.94, 95% confidence interval: 0.90–0.97, *P*_meta_ = 4.70 × 10^−9^). Importantly, we identified basophils and eosinophils that both exhibited a significantly decreased infiltration in colorectal tumors, and were regulated by rs4246215 through causal pathways involving both *FEN1* and DNA replication. In conclusion, this trans-omics incorporating GWAS data provides insights into a plausible pathway connecting DNA replication and immunity, expanding biological knowledge of colorectal tumorigenesis and therapeutic targets.

## Introduction

Colorectal cancer is among the top ten cancer types globally^[1]^, ranking the fourth in terms of incidence, and the second in terms of mortality in the United States as of 2022^[2]^. Both sporadic and hereditary forms of colorectal cancer arise from complex factors, such as dietary and environmental hazard exposure, as well as germline mutations (accounting for up to 6%–10% of all causes)^[3–4]^. A complete and accurate DNA replication is not only crucial for genomic stability but also a vulnerable cellular process that can initiate colorectal cancer and other cancers^[5–6]^.

The tumor immune microenvironment is a complex and dynamic entity that comprises various innate and adaptive immune cells, stromal cells, and extracellular matrix components^[7]^. It is noteworthy to mention that immune cells can exert either anti-cancer or pro-tumor effects through their interactions with tumor cells, and the spatial correlation between them can be investigated by multiplex immunofluorescence^[8–10]^. Although immunotherapy targeting immune cell receptors or ligands, such as programmed death-1, has revolutionized cancer treatment, the emergence of drug resistance remains an inevitable challenge^[11]^.

Recent advances in high-throughput technologies, including genomics, transcriptomics, epigenomics, and proteomics, have facilitated a comprehensive understanding of biological systems at multiple levels^[12]^. The integration of different omics approaches is a promising strategy that may facilitate a more detailed molecular understanding of health and disease^[13–14]^, helping in biomarker identification to clarify disease etiology and guide therapeutic development^[15–17]^.

In the current study, we performed integrated analyses of genomic, transcriptomic, and proteomic profiles to explore potential causal genetic loci, susceptibility genes, and biological pathways associated with colorectal cancer risk, as well as the association between DNA replication and immunity.

## Materials and methods

### Transcriptomic and proteomic profiles of colorectal cancer

The Nanjing Colorectal Cancer Cohort dataset, which is a long-term follow-up clinical cohort, provides transcriptomic data for 79 pairs of colorectal tumor tissues and adjacent normal tissues, as well as proteomic data for 25 paired tumors and adjacent normal tissues. The characteristics of participants and the recruitment protocols have been described previously^[18–20]^. Demographic characteristics are shown in ***Supplementary Table 1*** (available online).

A public dataset with information on 644 tumor tissues and 51 normal tissues was obtained from TCGA. Another three independent colorectal cancer datasets were obtained from GEO, which include GSE74602 (30 paired normal and tumor samples, Southeast Asian populations), GSE106582 (77 tumors and 117 mucosa tissues, European populations), and GSE117606 (74 tumors and 65 adjacent normal tissues, European populations).

### Genetic association study of colorectal cancer

The dataset of GWAS summary statistics of East Asian populations covered 7062 colorectal cancer cases and 195745 controls, with 8678297 single nucleotide polymorphisms (SNPs) (***Supplementary Table 1***). Details regarding genotyping and imputation were described elsewhere^[21]^. The qualified GWAS summary data were selected according to the following inclusion criteria: (1) call rate > 95%; (2) *P*-value for Hardy-Weinberg equilibrium (HWE) > 1 × 10^−6^; (3) minor allele frequency (MAF) > 0.05; and (4) *R*^*2*^ (imputation quality measure by Minimac3) > 0.80.

The GWAS summary data of European populations combined a sample size of 24476 cases and 23073 controls, with 6709910 SNPs. Detailed information about GECCO can be found in previous studies^[18,22–24]^. Demographic characteristics are shown in ***Supplementary Table 1***. The inclusion criteria for qualified GWAS summary data were as follows: (1) call rate > 95%; (2) MAF > 0.05; (3) *P*_HWE_ > 1×10^−6^; and (4) imputation accuracy *R*^*2*^ > 0.30.

### Pathway enrichment and activity estimation

Gene set enrichment analysis (GSEA)^[25]^ and gene set variation analysis (GSVA)^[26]^ were performed at the RNA and protein levels, based on the pathway in the Kyoto Encyclopedia of Genes and Genomes (KEGG) in Molecular Signatures Databases (MSigDB, http://www.gsea-msigdb.org/gsea/msigdb/index.jsp) *via* the R packages clusterProfiler^[27]^ and GSVA, respectively. We reserved enriched pathways from GSEA with a false discovery rate threshold of 0.05 for further analysis. The difference in pathway activity between tumor and normal samples calculated by GSVA was estimated by the R package limma^[28]^. We selected pathways from GSVA with an adjusted *P*-value cutoff of 0.05 and the absolute value of log_2_(fold change, FC) > 0.2^[29]^. To facilitate subsequent analysis, the resulting pathways were driven by intersecting the outcomes of GSEA and GSVA.

### Gene and gene-set analysis *via* genetic association study

Gene and gene-set analyses were performed by Multi-marker Analysis of GenoMic Annotation (MAGMA)^[30]^ using genome-wide summary statistics from East Asian and European populations. In the gene-based analysis, SNPs located between transcription start and stop sites of a gene were annotated to the gene, based on dbSNP version 135 SNP locations and NCBI 37.3 gene definitions, to assess the joint association of all SNPs in the gene with the phenotype. Similarly, in the gene-set analysis, individual genes were aggregated into biological pathways or cellular functions underlying MSigDB, showing the potential genetic etiology of phenotypes. The pathways met the criteria of having significant associations, as determined by a Bonferroni-adjusted *P*-value of less than 0.05, were selected along with their corresponding genes and SNPs. Comprehensive information regarding these pathways, genes, and SNPs can be found in ***Supplementary Tables 2*** and ***3*** (available online).

### Deconvolution of the tumor immune microenvironment at the transcriptome level

The tumor immune microenvironment was assessed by CIBERSORT^[31]^ and xCell^[32]^ algorithms using transcriptomic data. CIBERSORT (http://cibersort.stanford.edu/) provides an estimation of the abundance of immune cell types associated with the LM22 gene signature matrix. xCell (http://xCell.ucsf.edu/), a gene signature-based method, enables cell type enrichment analysis from gene expression data for 64 kinds of cell types, including innate and adaptive immune cells, hematopoietic progenitor cells, epithelial cells, and extracellular matrix cells. Detailed immune cell types are shown in ***Supplementary Table 4*** (available online).

### *In silico* analysis of genetic locus and functional annotation

Functional Annotation of Variants-Online Resource (FAVOR, http://favor.genohub.org/), a method providing functional annotations for 13 major categories, was used to annotate the selected SNPs and plot genetic functions. SAIGE UKB (https://pheweb.org/UKB-SAIGE/) supplied phenome-wide association study (PheWAS) summary statistics of the selected SNPs, which included 1403 ICD-based traits^[33]^. Region plots^[34]^ were generated by LocusZoom. Genotype and expression data (transcript and protein levels) were merged to perform expression quantitative trait loci (eQTL) in colorectal tumor and normal tissues in a linear regression model^[35]^. Details on genotype data collection, imputation, and processing have been published before^[19,36–37]^.

### Causal inference analysis of SNP, pathway, and immune features

The associations between the candidate variant and immune features underlying the causal pathway, mediated by gene expression/pathway activity, were assessed based on four scenarios: (1) merging all associations between genetic variants and immunity ($ {\beta }_{c} $); (2) merging all associations between genetic variants and gene expression/pathway activity ($ {\beta }_{a} $); (3) merging all associations between gene expression/pathway activity and immunity ($ {\beta }_{b} $); and (4) causal inference analysis. The indirect effect of genetic variants on immunity mediated by gene expression/pathway activity was estimated using the equation $ {\beta }_{indirect} = {\beta }_{a}$ × ${\,\beta }_{b}  $. The statistical *P*-value for indirect effects was determined using bootstrapping method^[38]^ *via* the R package mediation^[39]^. The correlation between flap structure-specific endonuclease 1 (*FEN1*) expression/pathway activity and immunity was analyzed by Spearman correlation underlying the R package psych^[40]^, and meta-analysis was performed *via* the R package metafor^[41]^.

### Multiplex immunofluorescence analysis

Multiplex immunofluorescence staining was performed at Servicebio Technology Co., Ltd. (Wuhan, China), and a multiplexed tyramide signal amplification method was used on 4 μm sections of the tissue microarray (TMA) containing 30 pairs of colorectal tumors and adjacent normal tissues from the Nanjing cohort. The details of TMA were reported previously^[22]^. After deparaffinization, antigen retrieval, and serum blocking, the TMA was sequentially stained with the primary antibodies anti-CD3 (GB13440, Servicebio), anti-CD45 (GB113885, Servicebio), anti-CD22 (66103-1-IG, Proteintech, Rosemont, IL, USA) and anti-FEN1 (14768-1-AP, Proteintech), followed by the appropriate secondary antibodies conjugated with horseradish peroxidase (HRP) and tyramine signal amplification (TSA) visualization. DAPI (4′,6-diamidino-2-phenylindole) was finally added to stain nuclei until all four markers were labeled. Antibody details are shown in ***Supplementary Table 5*** (available online). The slide was scanned with the Pannoramic MIDI (3DHISTECH, Hungary). Multispectral images were evaluated using the HALO Image Analysis Platform (Version 3.0.311.314, Indica, US). Basophils (CD22^+^), eosinophils (CD45^+^), T cells (CD3^+^) and FEN1-positive cells were quantified, and their average intensity in TMA was calculated.

### Statistical analysis

All statistical analyses were performed using R software (version 4.0.5). The normality of the variables was assessed using the Shapiro-Wilk test. The Chi-squared test, Fisher's exact test, Student's *t*-test, and Wilcoxon test were used as appropriate for the respective variables. Correlations between variables were assessed using either Spearman's or Pearson's correlation, depending on the normal distribution of the data. The false discovery rate correction was used in the RNA-seq analysis to manage false positives in multiple tests, efficiently pinpointing genes with significantly differential expression levels. In the SNP analysis with lower-dimensional genotype data, Bonferroni correction was applied to adjust the significance level for the number of comparisons conducted, reducing the likelihood of false discoveries. Bonferroni correction provides the most conservative results, emphasizing highly significant associations. A *P*-value less than 0.05, with a two-tailed test, was considered statistically significant. To perform the meta-analysis of the summary-level GWAS results for East Asian and European populations, we used METAL software, with the fixed-effects modeling and inverse-variance weighting^[42]^.

## Results

### Identification of differential biological pathways in colorectal tumors

**Figure 1:**
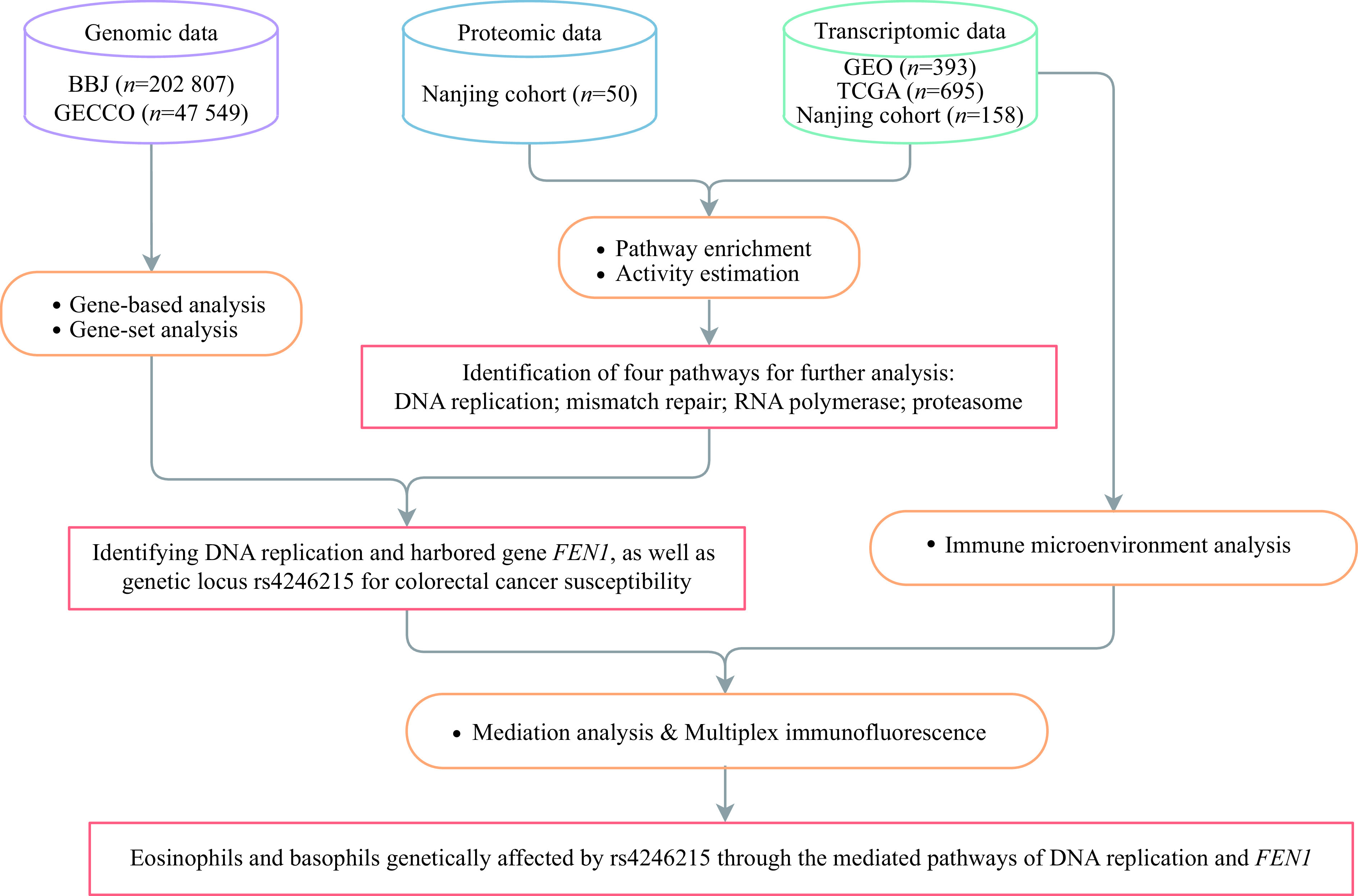
Flow chart of the study design.

**Figure 2:**
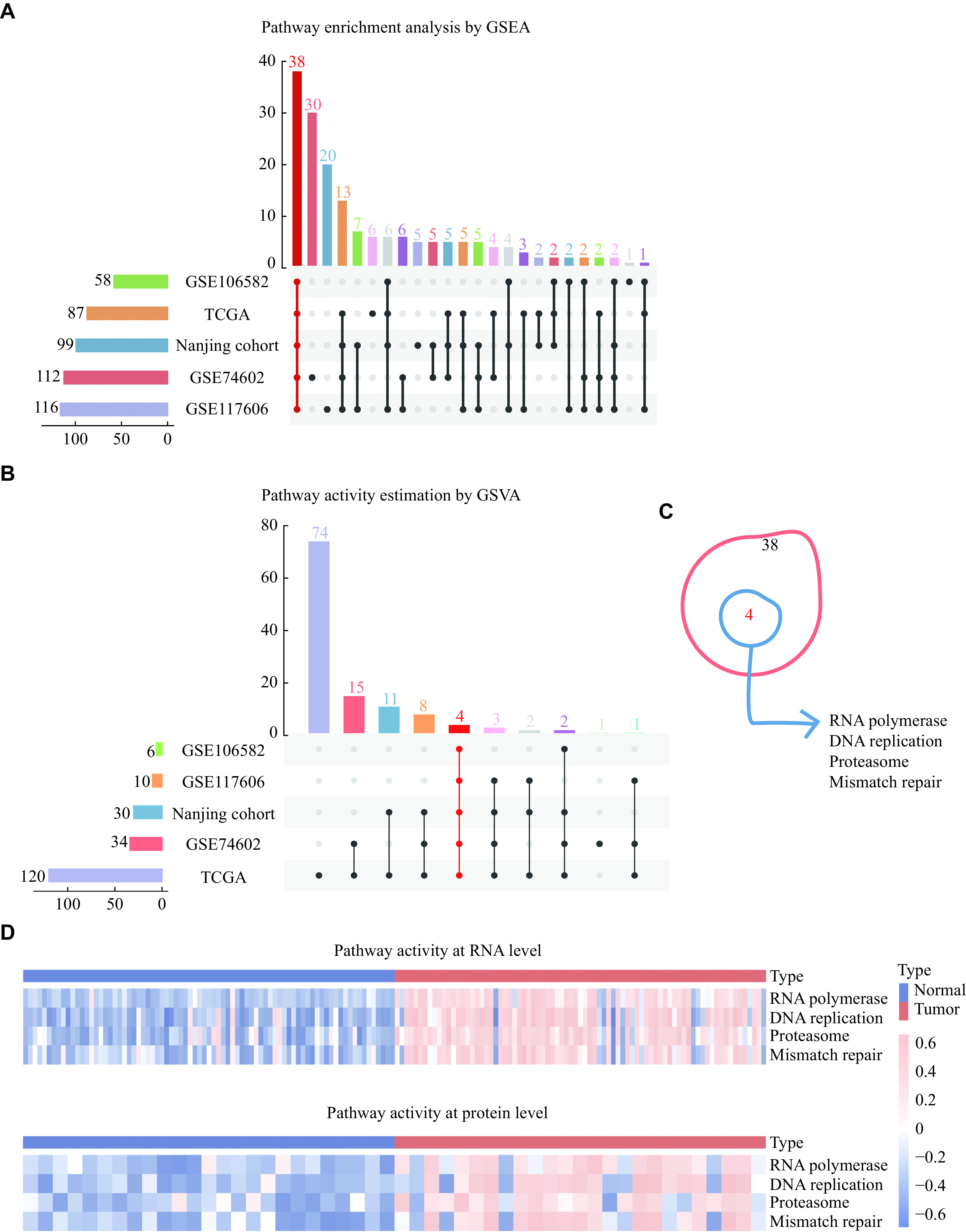
Pathway enrichment analysis and activity estimation at both the RNA and protein levels.

The flowchart of the current study is shown in ***Fig. 1***. We first analyzed the differentially enriched pathways between colorectal tumors and adjacient normal tissues using the GSEA method. Although some pathways were specifically enriched in individual datasets, 38 pathways were consistently enriched in colorectal tumors (***Fig. 2A***, ***Supplementary Table 6*** [available online]). We then used GSVA to estimate the pathway activity (***Supplementary Fig. 1A***–***1E***, available online) and found four pathways differentially enriched between colorectal tumors and adjacient normal tissues across all datasets (*i.e.*, DNA replication, mismatch repair, proteasome, and RNA polymerase; ***Fig. 2B***). Remarkably, these four pathways were the same as those identified by GSEA (***Fig. 2C***). Moreover, we performed GSEA (***Supplementary Table 7***, available online) and GSVA (***Supplementary Fig. 1F***, available online) on the Nanjing cohort proteome profiles, revealing that the four pathways enriched at the RNA level were also enriched at the protein level, thus being selected for further investigation (***Fig. 2D***).

### Estimation of genetic effects in pathways on colorectal cancer susceptibility

**Figure 3:**
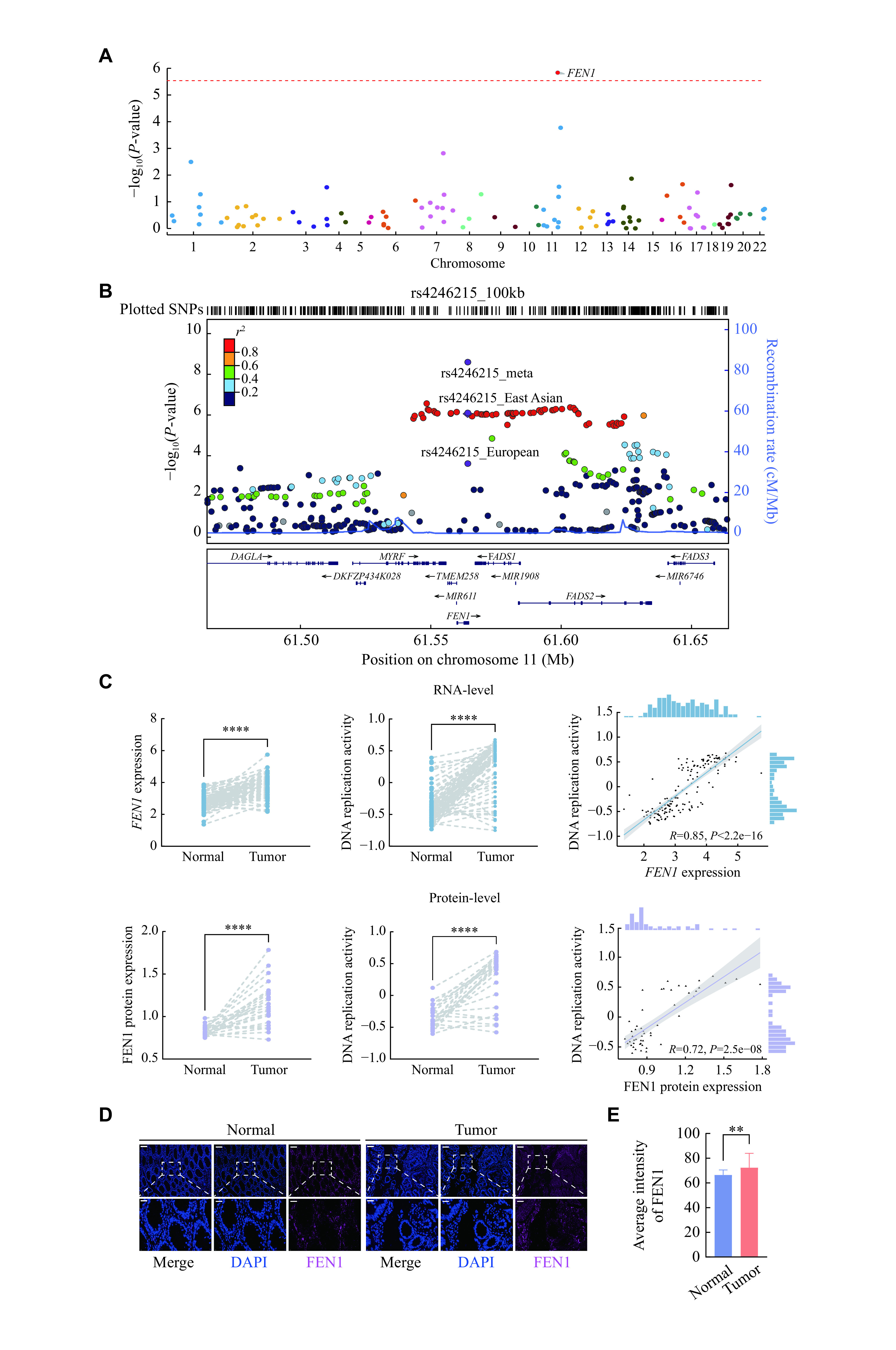
Genetic effects on colorectal cancer susceptibility and expression pattern of *FEN1* and DNA replication activity.

We then assessed genomic information on colorectal cancer susceptibility to determine key genes in the four candidate pathways. Interestingly, *FEN1*, one of 108 genes in the four selected pathways (specifically the DNA replication pathway), was significantly associated with colorectal cancer susceptibility in East Asian populations (*P*_Bonferroni_ = 0.025; ***Fig. 3A*** and ***Supplementary Table 2*** [available online]), and was subsequently validated in European populations (*P* = 4.41 × 10^−4^; ***Supplementary Table 2***). Variant-to-gene annotation (***Table 1***, ***Fig. 3B***) revealed that only rs4246215 at 11q12.2 in *FEN1* was associated with colorectal cancer susceptibility in both East Asian (OR = 0.92, 95% CI: 0.87–0.95, *P* = 1.46 × 10^−6^) and European (OR = 0.95, 95% CI: 0.92–0.98, *P* = 4.41 × 10^−4^) populations and reached genome-wide significance (OR = 0.94, 95% CI: 0.90–0.97, *P* = 4.70 × 10^−9^) without heterogeneity (*P*_het_ = 0.27, *I*^*2*^ = 0.20). However, no pathway passed the significance threshold of *P* < 0.05 (***Supplementary Table 3***, available online).

**Table 1:** Association between rs4246215 and colorectal cancer susceptibility in Asian and European populations

Variant	CHR	BP	Reference/ effect allele	Gene	Populations	EAF	OR (95% CI)	*P*	*P*_heterogeneity_	*I* ^*2*^
rs4246215	11	61564299	G/T	*FEN1*	East Asian	0.393	0.92 (0.87–0.95)	1.46×10^−6^		
					European	0.328	0.95 (0.92–0.98)	4.41×10^−4^		
					Combined		0.94 (0.90–0.97)	4.70×10^−9^	0.27	0.20
*P*-values of heterogeneity and meta-analysis were generated using METAL in fixed-effect inverse-variance. Abbreviations: CHR, chromosome; BP, base pair position in GRCh37/hg19; EAF, effect allele frequency; OR, odds ratio; CI, confidence interval.										

Functional annotation revealed that rs4246215 was located at the 3′-untranslated-region of *FEN1*, harboring strong functional signals of the enhancer activity, histone modification and transcription factor binding calculated by the FAVOR website (***Supplementary Table 8***, available online). PheWAS for pleiotropy evaluation *via* SAIGE UKB revealed that rs4246215 was dramatically associated with multiple phenotypes, especially in solid tumors and hematologic system tumors (***Supplementary Fig. 2A***, available online). Notably, both FEN1 expression (RNA level: log_2_FC = 0.32, *P* < 0.0001, Nanjing cohort; protein-level: log_2_FC = 0.45, *P* < 0.0001, Nanjing cohort) and DNA replication activity (RNA level: log_2_FC = 0.39, *P* = 1.40 × 10^−19^, Nanjing cohort; protein-level: log_2_FC = 0.34, *P* = 6.43 × 10^−5^, Nanjing cohort) were significantly increased in colorectal tumors and were positively associated with one another at both the RNA (*r* = 0.85, *P* < 2.20 × 10^−16^, Nanjing cohort) and protein (*r* = 0.72, *P* = 2.50 × 10^−8^, Nanjing cohort) levels (***Fig. 3C*** and ***Supplementary Fig. 3*** [available online]). Subsequent multiplex immunofluorescence staining confirmed the high expression of FEN1 in colorectal tumors (***Fig. 3D*** and ***3E***).

### Evaluation of immune responses in colorectal tumorigenesis

**Figure 4:**
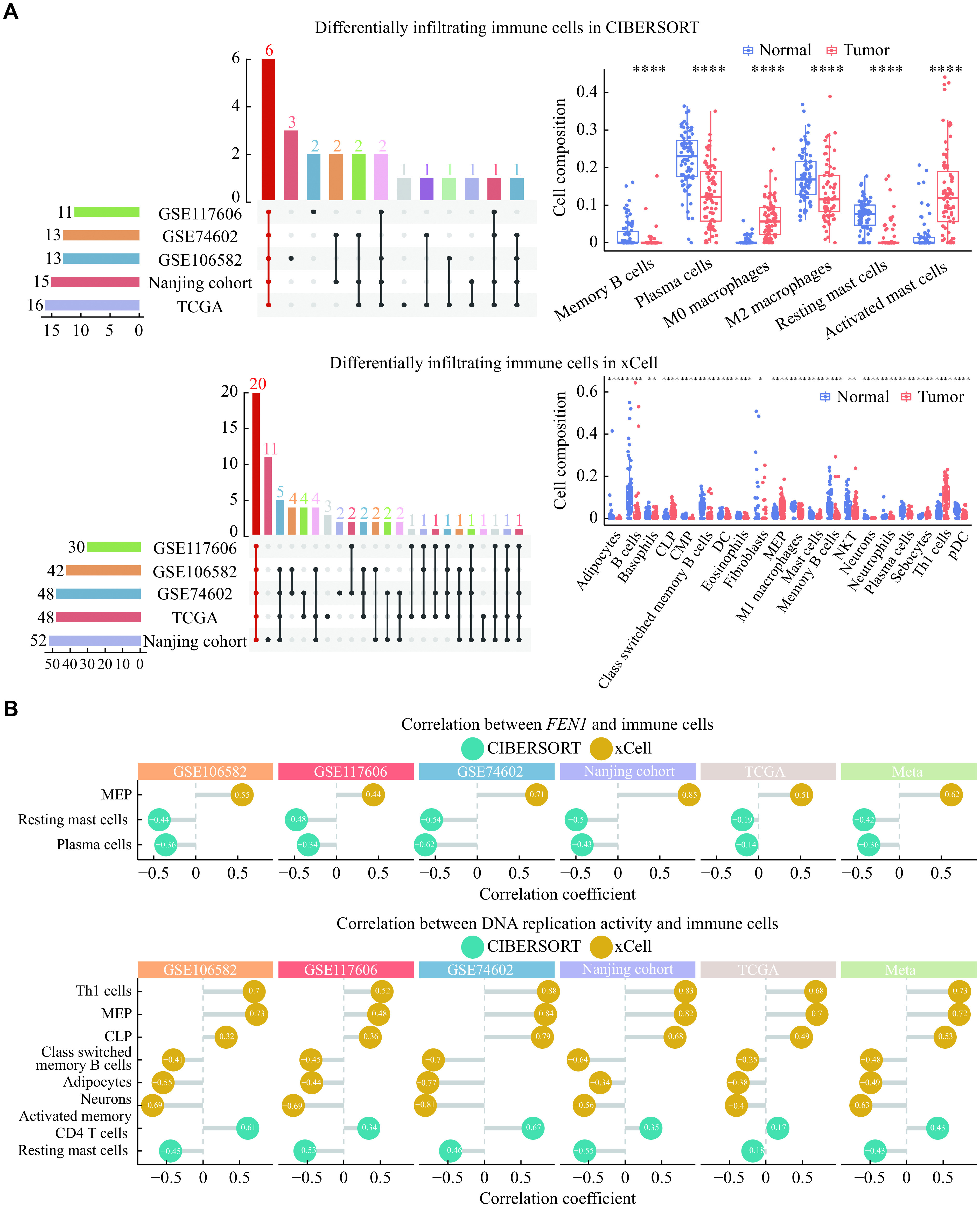
Differentially infiltrating immune cells and their correlations with *FEN1* and DNA replication.

We then assessed infiltrating immune cells in colorectal tumors and adjacient normal tissues to determine whether *FEN1* and DNA replication are involved in colorectal tumorigenesis *via* the immune pathway (***Supplementary Fig. 4***, available online). Across the five datasets, a total of 26 distinct immune cells were consistently identified, albeit some cell types being exclusively present in specific datasets (***Fig. 4A***). The proportions of most immune and stromal cells exhibited a decrease in colorectal tumors, but the proportions of Th1 cells, activated mast cells and M0 macrophages, showed an increase.

Furthermore, we conducted an additional analysis to determine the correlation of *FEN1* expression or DNA replication activity with the infiltration of immune cells. Interestingly, the infiltration levels of three immune cell types were significantly correlated with *FEN1*, and those of eight immune cell types were significantly correlated with DNA replication (***Fig. 4B***). Resting mast cells (*FEN1*: *r*_*meta*_ = −0.42, *P* < 0.0001; DNA replication: *r*_*meta*_ = −0.43, *P* < 0.0001) and megakaryocyte-erythroid progenitors (MEP; *FEN1*: *r*_*meta*_ = 0.62, *P* < 0.0001; DNA replication: *r*_*meta*_ = 0.72, *P* < 0.0001) exhibited consistent correlation patterns with *FEN1* expression and DNA replication activity.

### Evaluation of the effect of rs4246215 on *FEN1* expression, DNA replication and the tumor immune microenvironment

**Figure 5:**
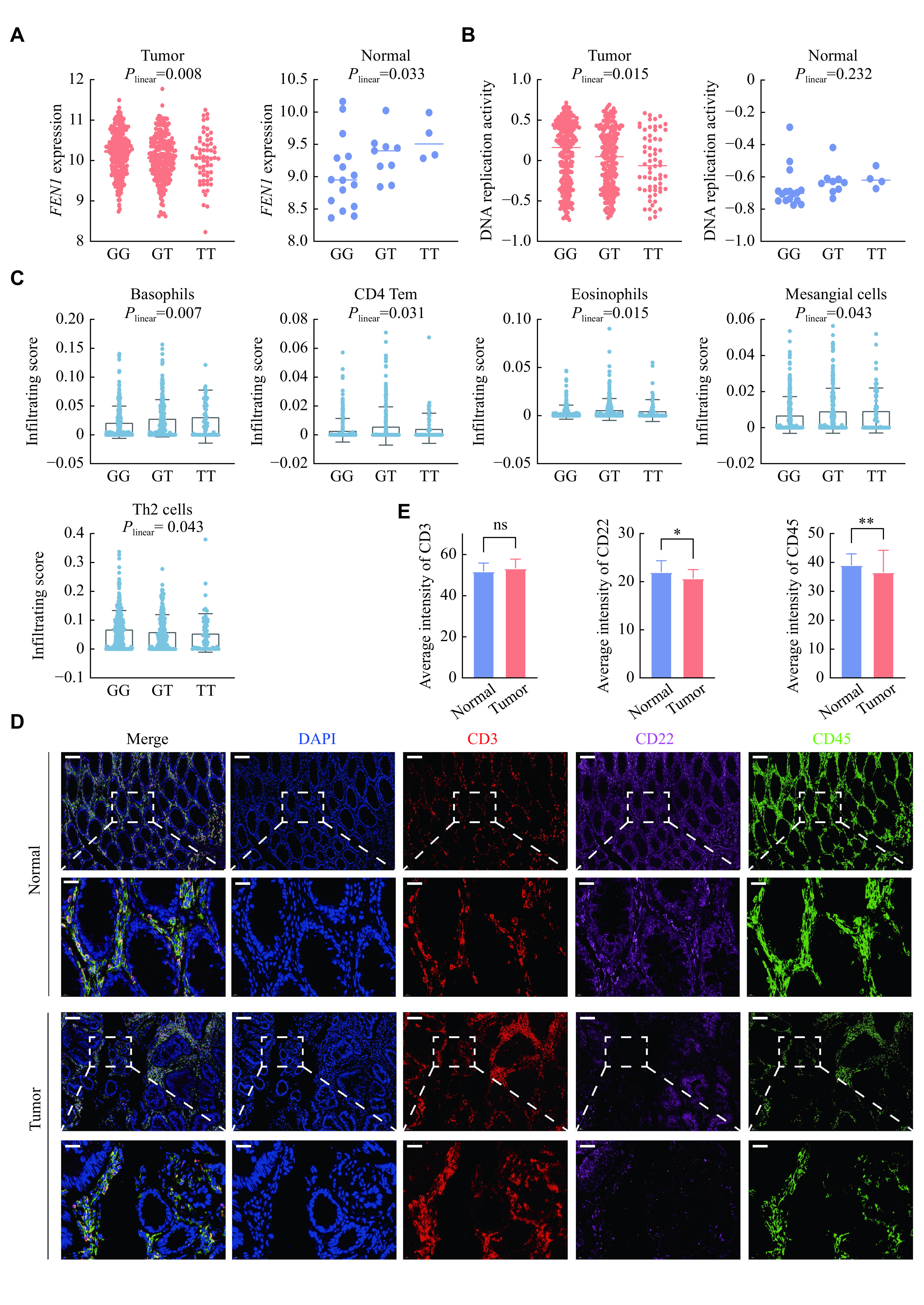
Genetic effect of rs4246215 and multiplex immunofluorescence evaluation.

The eQTL mapping was conducted to confirm the targets of rs4246215. Intriguingly, we observed that rs4246215 led to a downregulation of *FEN1* expression in colorectal tumors, while it conversely upregulated *FEN1* expression in normal tissues (***Fig. 5A***). In the Genotype-Tissue Expression Project (GTEx) database, a similar trend was observed in colon tissues (*β*_*sigmoid*_ = −0.003, *P* = 0.90; *β*_*transverse*_ = −0.004, *P* = 0.80), although the difference was not statistically significant. In addition, we found that rs4246215 decreased DNA replication activity in colorectal tumors but not in normal tissues (***Fig. 5B***) and dramatically influenced the proportions of five immune cells (*i.e.*, basophils, CD4 Tem, eosinophils, mesangial cells, and Th2 cells; ***Fig. 5C*** and ***Supplementary Table 9*** [available online]). However, the aforementioned trends were not significant in the Nanjing cohort (***Supplementary Fig. 2B***–***2D***, available online), potentially due to the limited sample size.

Subsequently, we performed multiplex immunofluorescence to visualize the colocalized biomarkers in the aforementioned immune cells (basophils, eosinophils and T cells) in the tumor immune microenvironment. Representative immunofluorescence staining of basophils, eosinophils and T cells, represented by CD22^+^, CD45^+^ and CD3^+^ staining, is shown in ***Fig. 5D***. In accordance with the immune infiltration analysis, the average levels of basophils (CD22^+^) and eosinophils (CD45^+^) infiltration in colorectal tumors were significantly lower than those in normal colorectal tissues (*P* = 0.017 and *P* = 0.003, respectively), but the difference in T cell (CD3^+^) infiltration levels was not statistically significant (***Fig. 5E***).

### Causal cascade of rs4246215, DNA replication, and immunity in colorectal cancer susceptibility

**Figure 6:**
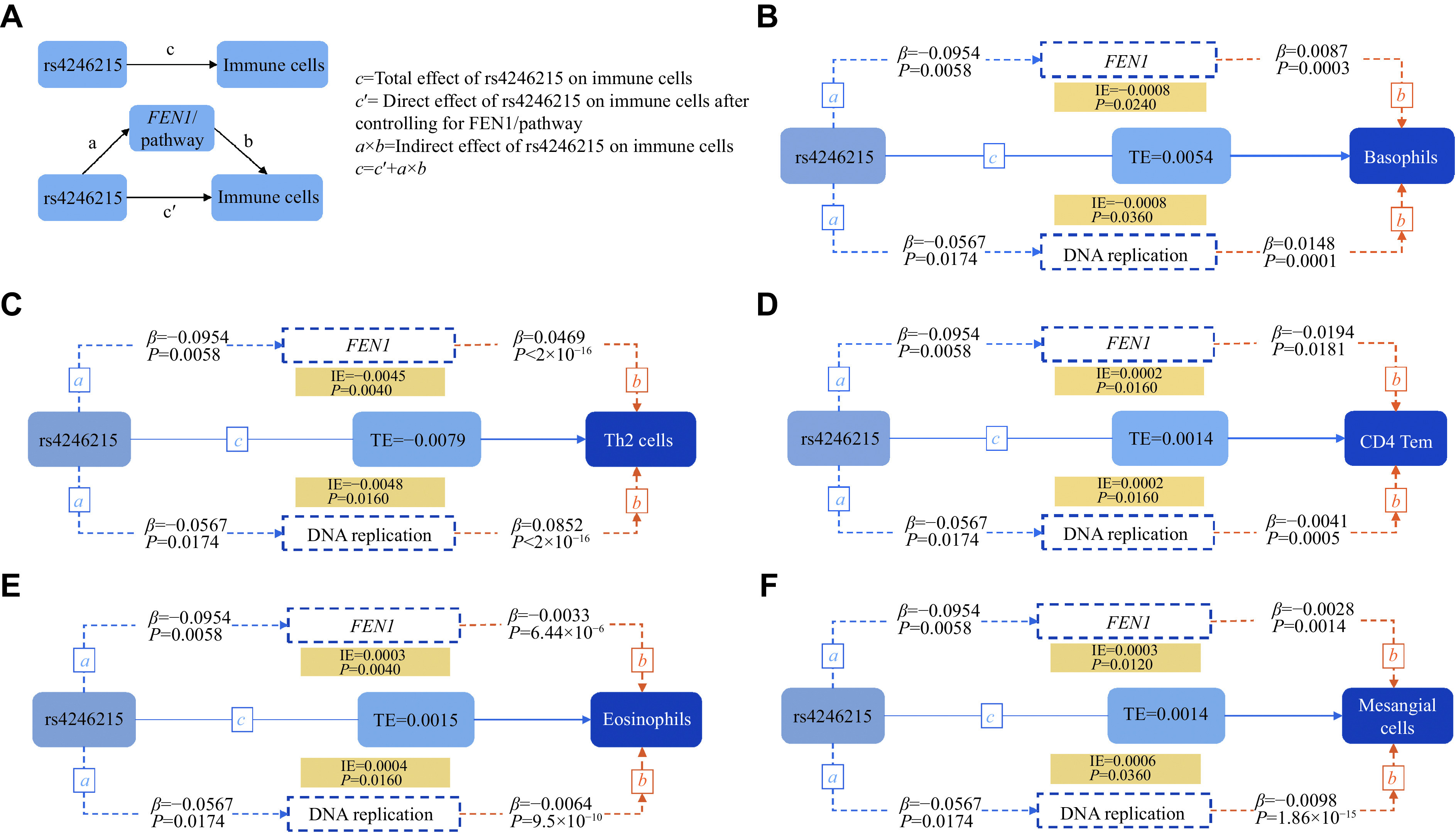
Causal mediation analysis between rs4246215 and immune cells mediated by *FEN1* and DNA replication.

We hypothesized that DNA replication and *FEN1* could act as mediators to influence the effect of rs4246215 on the immunity (***Fig. 6***). Upon the causal mediation analysis, we observed five significant indirect pathways, by which rs4246215 affects the immunity; both *FEN1* and DNA replication mediated the positive indirect effects of rs4246215 on CD4 Tem (*FEN1*: *β*_*indirect*_ = 0.0002, *P* = 0.016, 12.78% effects mediated; DNA replication: *β*_*indirect*_ = 0.0002, *P* = 0.016, 16.23% effects mediated), eosinophils (*FEN1*: *β*_*indirect*_ = 0.0003, *P* = 0.004, 21.42% effects mediated; DNA replication: *β*_*indirect*_ = 0.0004, *P* = 0.016, 24.83% effects mediated) and mesangial cells (*FEN1*: *β*_*indirect*_ = 0.0003, *P* = 0.012, 18.38% effects mediated; DNA replication: *β*_*indirect*_ = 0.0006, *P* = 0.036, 38.45% effects mediated), whereas they exerted negative indirect effects on basophils (*FEN1*: *β*_*indirect*_ = −0.0008, *P* = 0.024; DNA replication: *β*_*indirect*_ = −0.0008, *P* = 0.036) and Th2 cells (*FEN1: β*_*indirect*_ = −0.0045, *P* = 0.004, 56.40% effects mediated; DNA replication: *β*_*indirect*_ = −0.0048, *P* = 0.016, 60.95% effects mediated).

## Discussion

In the current study, we investigated the potential causal protective locus rs4246215 G>T in *FEN1*, a locus associated with DNA replication, and its effect on the colorectal tumor immune microenvironment involved in tumorigenesis.

Obstacles that impede DNA replication can lead to DNA replication stress, a phenomenon causing genetic mutations and instability^[43]^. Chronic DNA replication stress is a hallmark of cancer cells, which provides a potential therapeutic target for cancer treatment^[44]^. Our findings highlight the significance of DNA replication in colorectal cancer and its potential as a therapeutic target. *FEN1*, a member of the DNA replication pathway, is also involved in other DNA metabolic pathways, including telomere stability maintenance and apoptotic DNA fragmentation^[45–46]^. *In vivo* and *in vitro* studies have demonstrated that *FEN1* is frequently overexpressed in various cancers, such as breast cancer, glioma, and hepatocellular cancer, and that its upregulation promotes tumorigenesis and cancer progression^[47–50]^. Additionally, *FEN1* overexpression can lead to genome instability and impair DNA replication through its interaction with PCNA^[51]^. We observed consistent FEN1 overexpression in colorectal tumors at both the RNA and protein levels, suggesting that FEN1 may serve as a promising biomarker for the diagnosis of colorectal cancer.

Previous studies have reported an association between rs4246215 and risk of various solid tumors, including cancers of lung, breast, and colorectum^[52]^. The current study adds to the existing knowledge by demonstrating the functional implications of rs4246215 in colorectal tumor immune microenvironment. Future investigation is warranted to elucidate functional implications of other potential SNPs and their interplay with rs4246215, to provide a better understanding of the genetic mechanisms underlying colorectal cancer pathagenesis.

Tumor immune microenvironment consists of tumor cells and various non-tumor cells and plays a critical role in tumor initiation, progression, and response to therapy^[53]^. In the current study, we employed the CIBERSORT and xCell algorithms to investigate the differential infiltration of immune cells in colorectal tumors, compared with normal tissues. Our results indicated that macrophages, B cells, and mast cells were differentially infiltrated in colorectal tumors. Macrophages, including M1 and M2 polarized macrophages, have been identified as potential immunotherapeutic targets^[54–55]^. B cells are significant prognostic factors for cancer, although the mechanisms underlying their immune-suppressive or immune-supportive effects remain poorly understood^[56–59]^. In addition, little is known about B cells as a new target for immunotherapy^[60–61]^. Meanwhile, the role of mast cell in the tumor immune microenvironment remains controversial, with contradictory results from different studies^[62]^. These findings highlight the potential of macrophages and B cells as immunotherapeutic targets and underscore the need for further research to elucidate their precise roles in immunotherapy of colorectal cancer.

Recent studies have demonstrated that targeting DNA replication stress can modulate the immune microenvironment and improve immunotherapy efficacy^[63]^. The current study contributes to this knowledge by revealing the intricate interplay among genetic variants, DNA replication, and the tumor immune microenvironment in colorectal cancer development. Investigating genetic variants that influence the infiltration of immune cells at the genomic level may provide new insights into the regulation of immune microenvironments and facilitate the development of novel immunotherapeutic strategies.

Multiplex immunofluorescence is a powerful tool to study the spatial tumor immune microenvironment of limited tissue specimens, which improves the understanding of tumor-immune interactions^[64–65]^. We validated our bioinformatic findings by using multiplex immunofluorescence to quantify immune cells and DNA replication activity in colorectal tumor and normal tissues at the protein level. Our results confirmed the spatial distribution of immune cells and tumor cells, and revealed differential infiltration of basophils, eosinophils, and FEN1 in colorectal tumors, compared with normal tissues. However, further studies are needed to better characterize other immune cell components in the colorectal tumor immune microenvironment.

It is important to acknowledge the limitations of the current study. We did not conduct *in vitro* and *in vivo* experiments to validate the differentially expressed genes found in the tumors and immune cell components. Future experiments should investigate immune-mediated cytotoxicity as a crucial mechanism in the tumor microenvironment. Additionally, the sample size of the cohort used to validate the data from public datasets was relatively small. Large-scale omics datasets are needed to confirm our results. Moreover, the current study focused on one SNP locus, and future studies should investigate other potential SNPs and their effects on immune microenvironment.

In summary, the current study uncovers the functional involvement of *FEN1* in colorectal cancer susceptibility through the DNA replication process and highlights the effect of the causal variant rs4246215 G>T on the colorectal tumor immune microenvironment. The current study contributes to the understanding of the pathogenesis of colorectal cancer and sheds light on the "SNP-gene/pathway-immunity" scheme that may have implications for clinical practice.

## SUPPLEMENTARY DATA

Supplementary data to this article can be found online.

## References

[1] (2021). Global Cancer Statistics 2020: GLOBOCAN estimates of incidence and mortality worldwide for 36 cancers in 185 countries. CA Cancer J Clin.

[2] (2022). Cancer statistics, 2022. CA Cancer J Clin.

[3] (2017). Cancer susceptibility gene mutations in individuals with colorectal cancer. J Clin Oncol.

[4] (2020). An update on colorectal cancer microenvironment, epigenetic and immunotherapy. Int Immunopharmacol.

[5] (2019). DNA replication stress and its impact on chromosome segregation and tumorigenesis. Semin Cancer Biol.

[6] (2015). DNA replication stress as a hallmark of cancer. Annu Rev Pathol.

[7] (2020). The tumor microenvironment. Curr Biol.

[8] (2019). The tumor microenvironment innately modulates cancer progression. Cancer Res.

[9] (2020). Overview of multiplex immunohistochemistry/immunofluorescence techniques in the era of cancer immunotherapy. Cancer Commun (Lond).

[10] (2020). The Society for Immunotherapy of Cancer statement on best practices for multiplex immunohistochemistry (IHC) and immunofluorescence (IF) staining and validation. J Immunother Cancer.

[11] (2021). Tumor microenvironment as a therapeutic target in cancer. Pharmacol Ther.

[12] (2019). Integrated omics: tools, advances, and future approaches. J Mol Endocrinol.

[13] (2021). Multi-omics integration in biomedical research - A metabolomics-centric review. Anal Chim Acta.

[14] (2018). Integrative omics for health and disease. Nat Rev Genet.

[15] (2021). Integrative omics provide biological and clinical insights into acute respiratory distress syndrome. Intensive Care Med.

[16] (2019). Integrated proteogenomic characterization of HBV-related hepatocellular carcinoma. Cell.

[17] (2021). An integrative analysis of the age-associated multi-omic landscape across cancers. Nat Commun.

[18] (2019). Combinations of single nucleotide polymorphisms identified in genome-wide association studies determine risk for colorectal cancer. Int J Cancer.

[19] (2016). Common genetic variation in *ETV6* is associated with colorectal cancer susceptibility. Nat Commun.

[20] (2022). Exosomal circLPAR1 functions in colorectal cancer diagnosis and tumorigenesis through suppressing *BRD4*
*via* METTL3-eIF3h interaction. Mol Cancer.

[21] (2020). Large-scale genome-wide association study in a Japanese population identifies novel susceptibility loci across different diseases. Nat Genet.

[22] (2022). Genome-wide association analyses identify CATSPERE as a mediator of colorectal cancer susceptibility and progression. Cancer Res.

[23] (2019). Discovery of common and rare genetic risk variants for colorectal cancer. Nat Genet.

[24] (2013). Identification of genetic susceptibility loci for colorectal tumors in a genome-wide meta-analysis. Gastroenterology.

[25] (2005). Gene set enrichment analysis: a knowledge-based approach for interpreting genome-wide expression profiles. Proc Natl Acad Sci U S A.

[26] (2013). GSVA: gene set variation analysis for microarray and RNA-seq data. BMC Bioinformatics.

[27] (2012). clusterProfiler: an R package for comparing biological themes among gene clusters. OMICS.

[28] (2004). Linear models and empirical Bayes methods for assessing differential expression in microarray experiments. Stat Appl Genet Mol Biol.

[29] (2006). The MicroArray Quality Control (MAQC) project shows inter- and intraplatform reproducibility of gene expression measurements. Nat Biotechnol.

[30] (2015). MAGMA: generalized gene-set analysis of GWAS data. PLoS Comput Biol.

[31] (2015). Robust enumeration of cell subsets from tissue expression profiles. Nat Methods.

[32] (2017). xCell: digitally portraying the tissue cellular heterogeneity landscape. Genome Biol.

[33] (2014). Detection of pleiotropy through a Phenome-wide association study (PheWAS) of epidemiologic data as part of the Environmental Architecture for Genes Linked to Environment (EAGLE) study. PLoS Genet.

[34] (2010). LocusZoom: regional visualization of genome-wide association scan results. Bioinformatics.

[35] (2012). Matrix eQTL: ultra fast eQTL analysis *via* large matrix operations. Bioinformatics.

[36] (2018). Circadian clock pathway genes associated with colorectal cancer risk and prognosis. Arch Toxicol.

[37] (2021). Systematic evaluation of the effects of genetic variants on PIWI-interacting RNA expression across 33 cancer types. Nucleic Acids Res.

[38] (2008). Asymptotic and resampling strategies for assessing and comparing indirect effects in multiple mediator models. Behav Res Methods.

[39] (2014). Mediation: R package for causal mediation analysis. J Stat Software.

[b40] 40Revelle W. Psych: procedures for psychological, psychometric, and personality research[EB/OL]. [2023-04-01] https://CRAN.R-project.org/package=psych.

[41] (2010). Conducting meta-analyses in R with the metafor package. J Stat Software.

[42] (2010). METAL: fast and efficient meta-analysis of genomewide association scans. Bioinformatics.

[43] (2022). Hallmarks of DNA replication stress. Mol Cell.

[44] (2019). Exploiting DNA replication stress for cancer treatment. Cancer Res.

[45] (2003). CRN-1, a *Caenorhabditis elegans* FEN-1 homologue, cooperates with CPS-6/EndoG to promote apoptotic DNA degradation. EMBO J.

[46] (2008). Flap endonuclease 1 contributes to telomere stability. Curr Biol.

[47] (2020). Endonuclease FEN1 coregulates ERα activity and provides a novel drug interface in tamoxifen-resistant breast cancer. Cancer Res.

[48] (2017). FEN1 promotes tumor progression and confers cisplatin resistance in non-small-cell lung cancer. Mol Oncol.

[49] (2022). Flap endonuclease 1 and DNA-PKcs synergistically participate in stabilizing replication fork to encounter replication stress in glioma cells. J Exp Clin Cancer Res.

[50] (2022). Flap endonuclease 1 facilitated hepatocellular carcinoma progression by enhancing USP7/MDM2-mediated P53 inactivation. Int J Biol Sci.

[51] (2018). Flap endonuclease overexpression drives genome instability and DNA damage hypersensitivity in a PCNA-dependent manner. Nucleic Acids Res.

[52] (2019). Association between the flap endonuclease 1 gene polymorphisms and cancer susceptibility: an updated meta-analysis. J Cell Biochem.

[53] (2013). The continuum of cancer immunosurveillance: prognostic, predictive, and mechanistic signatures. Immunity.

[54] (2020). Engineering macrophages for cancer immunotherapy and drug delivery. Adv Mater.

[55] (2021). Macrophage-based approaches for cancer immunotherapy. Cancer Res.

[56] (2012). The prognostic significance of B lymphocytes in invasive carcinoma of the breast. Breast Cancer Res Treat.

[57] (2019). The prognostic importance of CD20^+^ B lymphocytes in colorectal cancer and the relation to other immune cell subsets. Sci Rep.

[58] (2020). Tertiary lymphoid structures improve immunotherapy and survival in melanoma. Nature.

[59] (2017). Interaction between tumour-infiltrating B cells and T cells controls the progression of hepatocellular carcinoma. Gut.

[60] (2019). Tumor-infiltrating B cells: their role and application in anti-tumor immunity in lung cancer. Cell Mol Immunol.

[61] (2020). Tertiary lymphoid structures and B cells: clinical impact and therapeutic modulation in cancer. Semin Immunol.

[62] (2018). Low tumor infiltrating mast cell density confers prognostic benefit and reflects immunoactivation in colorectal cancer. Int J Cancer.

[63] (2021). Targeting KDM4A epigenetically activates tumor-cell-intrinsic immunity by inducing DNA replication stress. Mol Cell.

[64] (2021). Multi-institutional TSA-amplified multiplexed immunofluorescence reproducibility evaluation (MITRE) study. J Immunother Cancer.

[65] (2020). Three-dimensional histologic, immunohistochemical, and multiplex immunofluorescence analyses of dynamic vessel co-option of spread through air spaces in lung adenocarcinoma. J Thorac Oncol.

