# Supplementary material for: Causal genetic regulation of DNA replication on immune microenvironment in colorectal tumorigenesis: Evidenced by an integrated approach of trans-omics and GWAS
Source: J Biomed Res. 2023 Dec 18;38(1):37–50. doi: 10.7555/JBR.37.20230081 (PMC10818172; doi:10.7555/JBR.37.20230081)
Supplement: Supplementary file 1 — Supplementary data to this article can be found online. [file jbr-38-1-37-S1.pdf]

Available online at www.jbr-pub.org.cn

Open Access at PubMed Central

The Journal of Biomedical Research, 2023 37(0): 1–17

Supplementary Data

JBR

Causal genetic regulation of DNA replication on immune microenvironment in colorectal tumorigenesis: evidenced by an integrated approach of trans-omics and GWAS

Sumeng Wang[1,△], Silu Chen[2,△], Huiqin Li[4], Shuai Ben[2,3], Tingyu Zhao[1], Rui Zheng[2,3], Meilin Wang[2,3], Dongying Gu[5,✉], Lingxiang Liu[1,✉]

[1]Department of Oncology, the First Affiliated Hospital of Nanjing Medical University, Nanjing, Jiangsu 210029, China;
[2]Department of Environmental Genomics, Jiangsu Key Laboratory of Cancer Biomarkers, Prevention and Treatment, Collaborative Innovation Center for Cancer Personalized Medicine, Nanjing Medical University, Nanjing, Jiangsu 211166, China;
[3]Department of Genetic Toxicology, the Key Laboratory of Modern Toxicology of Ministry of Education, Center for Global Health, School of Public Health, Nanjing Medical University, Nanjing, Jiangsu 211166, China;
[4]Department of Biostatistics, Center for Global Health, School of Public Health, Nanjing Medical University, Nanjing, Jiangsu 211166, China;
[5]Department of Oncology, Nanjing First Hospital, Nanjing Medical University, Nanjing, Jiangsu, 210006, China; △The authors contributed equally.

Supplementary Table 1 **Demographic characteristics of individuals in each cohort**

Variables	Nanjing colorectal cancer cohort		East Asian populations (BBJ)		European populations (GECCO)	
	Transcriptome	Proteome	Cases	Controls	Cases	Controls
	n=79	*n*=25	*n*=7 062	*n*=195 745	*n*=24 476	*n*=23 073
Age (mean ± SD)	58.2 ± 16.7	67.6 ± 12.2	67.0 ± 10.2	61.6 ± 13.9	63.8 ± 10.4	60.1 ± 11.5
Sex [*n* (%)]						
Male	42 (53.2)	15 (60.0)	4 496 (63.7)	97 655 (49.9)	13 017 (53.2)	11 084 (48.0)
Female	37 (46.8)	10 (40.0)	2 566 (36.3)	98 090 (50.1)	11 459 (46.8)	11 989 (52.0)

Abbreviation: SD, standard deviation.

△The authors contributed equally.

✉Corresponding authors: Dongying Gu, Department of Oncology, Nanjing First Hospital, Nanjing Medical University, 68 Changle Road, Nanjing, Jiangsu 210006, China. E-mail: dygu@njmu.edu.cn; Lingxiang Liu, Department of Oncology, the First Affiliated Hospital of Nanjing Medical University, 300 Guangzhou Road, Nanjing, Jiangsu, 210029, China. E-mail: llxlau@163.com.

Received: 07 April 2023; Revised: 26 May 2023; Accepted: 28 May 2023; Published online: 18 December 2023

CLC number: R735.3, Document code: A

The authors reported no conflict of interests.

This is an open access article under the Creative Commons Attribution (CC BY 4.0) license, which permits others to distribute, remix, adapt and build upon this work, for commercial use, provided the original work is properly cited.

© 2023 by the Journal of Biomedical Research.

https://doi.org/10.7555/JBR.37.20230081

Supplementary Table 2 **Detailed information about genes on four pathways annotated by MAGMA. *FEN1* is associated with colorectal cancer susceptibility both in East Asian and European populations. Genes on four pathways annotated by MAGMA in Biobank JAPAN but not in GECCO were left in blank**

Pathway	Gene	CHR	Start	Stop	Biobank Japan							GECCO						
					NSNPS	NPARAM	*N*	ZSTAT	*P*	*FDR*	Bonferroni	NSNPS	NPARAM	*N*	ZSTAT	*P*	*FDR*	Bonferroni
**DNA replication**	*FEN1*	11	61 560 109	61 564 716	1	1	202 807	4.68	1.46E−06	2.29E−03	**2.51E−02**	1	1	45 431	3.33	**4.41E−04**	8.49E−02	1
	MCM7	7	99 690 351	99 699 563	8	2	202 807	2.96	1.52E−03	2.43E−01	1	9	3	47 396	2.35	9.34E−03	3.38E−01	1
	POLD1	19	50 887 531	50 921 275	51	2	202 807	1.98	2.37E−02	5.77E−01	1	34	1	46 993	0.67	2.52E−01	7.77E−01	1
	RNASEH2C	11	65 485 144	65 488 409	3	2	202 807	1.51	6.54E−02	6.80E−01	1	3	1	47 237	−0.81	7.90E−01	9.53E−01	1
	DNA2	10	70 173 821	70 231 878	154	7	202 807	1.02	1.53E−01	7.97E−01	1	126	7	47 246	1.16	1.23E−01	6.62E−01	1
	RPA1	17	1 733 273	1 802 848	172	15	202 807	0.96	1.69E−01	8.07E−01	1	156	13	47 081	0.39	3.49E−01	8.24E−01	1
	PRIM1	12	57 125 364	57 146 146	14	1	202 807	0.91	1.81E−01	8.15E−01	1	13	1	45 999	0.54	2.94E−01	7.92E−01	1
	MCM5	22	35 796 116	35 820 495	49	7	202 807	0.83	2.03E−01	8.30E−01	1	47	9	46 803	−0.78	7.84E−01	9.53E−01	1
	RFC1	4	39 289 069	39 368 001	91	4	202 807	0.61	2.71E−01	8.70E−01	1	85	5	46 716	2.06	1.97E−02	4.20E−01	1
	LIG1	19	48 618 702	48 673 852	182	10	202 807	0.52	3.02E−01	8.83E−01	1	147	8	47 219	2.15	1.59E−02	3.95E−01	1
	RPA2	1	28 218 035	28 241 255	44	2	202 807	0.45	3.28E−01	8.99E−01	1	34	2	47 058	0.44	3.29E−01	8.12E−01	1
	POLD2	7	44 154 279	44 163 267	13	3	202 807	0.37	3.55E−01	9.10E−01	1	7	3	46 308	−0.61	7.29E−01	9.38E−01	1
	POLE2	14	50 110 270	50 155 098	5	1	202 807	0.30	3.84E−01	9.18E−01	1	69	2	47 348	−0.72	7.63E−01	9.46E−01	1
	RNASEH1	2	3 592 675	3 605 957	24	4	202 807	0.25	4.02E−01	9.19E−01	1	25	6	46 948	0.12	4.53E−01	8.69E−01	1
	MCM4	8	48 872 763	48 890 720	5	1	202 807	0.17	4.33E−01	9.24E−01	1	5	1	47 216	−0.42	6.64E−01	9.23E−01	1
	RNASEH2B	13	51 483 814	51 544 596	57	5	202 807	−0.11	5.42E−01	9.48E−01	1	72	9	46 975	0.30	3.81E−01	8.40E−01	1
	RFC3	13	34 392 206	34 540 695	297	13	202 807	−0.16	5.65E−01	9.54E−01	1	159	9	47 268	1.58	5.75E−02	5.47E−01	1
	POLA2	11	65 029 432	65 065 088	29	4	202 807	−0.21	5.84E−01	9.57E−01	1	21	2	47 318	−1.09	8.61E−01	9.68E−01	1
	RFC4	3	186 507 681	186 524 484	39	4	202 807	−0.69	7.54E−01	9.80E−01	1	23	3	46 986	−0.07	5.27E−01	8.90E−01	1
	MCM6	2	136 597 196	136 634 047	93	9	202 807	−0.72	7.64E−01	9.80E−01	1	20	3	47 079	−0.55	7.09E−01	9.35E−01	1
	POLE	12	133 200 344	133 264 050	148	5	202 807	−0.89	8.13E−01	9.88E−01	1	150	6	47 410	−0.61	7.28E−01	9.38E−01	1
	POLE4	2	75 185 775	75 196 859	15	2	202 807	−0.93	8.24E−01	9.88E−01	1	14	3	47 142	−1.34	9.09E−01	9.74E−01	1
	MCM2	3	127 317 200	127 341 279	14	4	202 807	−1.05	8.52E−01	9.91E−01	1	15	4	46 859	−0.60	7.25E−01	9.38E−01	1
	RPA3	7	7 676 347	7 758 238	197	12	202 807	−1.42	9.23E−01	9.98E−01	1	223	16	47 067	−1.34	9.10E−01	9.75E−01	1
	RNASEH2A	19	12 912 863	12 924 462	1	1	202 807	−1.62	9.47E−01	1.00E+00	1	8	3	46 272	−0.40	6.55E−01	9.22E−01	1
	MCM3	6	52 128 812	52 149 679	52	7	202 807	−1.73	9.58E−01	1.00E+00	1	45	7	47 256	−1.09	8.62E−01	9.68E−01	1

Supplementary Table 2 **Detailed information about genes on four pathways annotated by MAGMA. *FEN1* is associated with colorectal cancer susceptibility both in East Asian and European populations. Genes on four pathways annotated by MAGMA in Biobank JAPAN but not in GECCO were left in blank** (continued)

Pathway	Gene	CHR	Start	Stop	Biobank Japan							GECCO						
					NSNPS	NPARAM	*N*	ZSTAT	*P*	*FDR*	Bonferroni	NSNPS	NPARAM	*N*	ZSTAT	*P*	*FDR*	Bonferroni
Mismatch repair	*POLD3*	11	74 303 575	74 380 386	169	9	202 807	3.58	1.69E−04	7.09E−02	1	188	13	47 254	5.01	2.76E−07	3.95E−04	4.73E−03
	POLD4	11	67 118 236	67 121 067	3	1	202 807	1.92	2.74E−02	5.95E−01	1	1	1	47 187	0.91	1.80E−01	7.19E−01	1
	MSH6	2	48 010 221	48 034 092	9	3	202 807	0.98	1.64E−01	8.05E−01	1	60	8	46 942	0.45	3.25E−01	8.10E−01	1
	RFC2	7	73 645 832	73 668 788	17	2	202 807	0.98	1.64E−01	8.05E−01	1	18	2	47 310	0.74	2.29E−01	7.63E−01	1
	PMS2	7	6 012 870	6 048 737	62	8	202 807	0.97	1.67E−01	8.06E−01	1	64	9	46 782	−0.70	7.58E−01	9.45E−01	1
	SSBP1	7	141 438 121	141 450 288	40	5	202 807	0.80	2.12E−01	8.34E−01	1	10	1	46 215	1.31	9.50E−02	6.20E−01	1
	RFC5	12	118 454 506	118 470 044	29	4	202 807	0.75	2.28E−01	8.47E−01	1	21	4	47 090	−0.39	6.52E−01	9.21E−01	1
	MLH1	3	37 034 841	37 092 337	51	2	202 807	0.69	2.45E−01	8.53E−01	1	49	2	46 397	2.85	2.20E−03	1.87E−01	1
	PCNA	20	5 095 599	5 107 268	1	1	202 807	0.18	4.30E−01	9.24E−01	1	14	1	46 835	−0.63	7.35E−01	9.40E−01	1
	EXO1	1	242 011 491	242 053 241	166	12	202 807	−0.24	5.94E−01	9.60E−01	1	100	6	47 142	−1.34	9.10E−01	9.75E−01	1
	MSH3	5	79 950 467	80 172 634	326	12	202 807	−0.25	6.00E−01	9.60E−01	1	519	18	47 199	1.08	1.39E−01	6.85E−01	1
	MSH2	2	47 630 206	47 739 716	159	7	202 807	−1.20	8.85E−01	9.94E−01	1	177	11	47 228	1.37	8.57E−02	6.00E−01	1
	MLH3	14	75 480 467	75 518 235	35	1	202 807	−2.46	9.93E−01	1.00E+00	1	36	2	46 630	1.26	1.04E−01	6.37E−01	1
Proteasome	*PSMA5*	1	109 941 653	109 969 108	4	1	202 807	2.73	3.21E−03	3.31E−01	1	25	3	46 754	−0.66	7.46E−01	9.42E−01	1
	PSMA3	14	58 711 523	58 738 727	22	1	202 807	2.21	1.36E−02	5.01E−01	1	29	3	47 273	−0.48	6.84E−01	9.27E−01	1
	PSMB10	16	67 968 407	67 970 780	1	1	202 807	2.01	2.21E−02	5.68E−01	1	1	1	46 481	−1.79	9.63E−01	9.86E−01	1
	PSMB3	17	36 908 978	36 920 484	14	4	202 807	1.70	4.50E−02	6.57E−01	1	17	5	46 405	−2.15	9.84E−01	9.94E−01	1
	PSMD4	1	151 227 176	151 239 955	12	1	202 807	1.62	5.24E−02	6.76E−01	1	11	2	47 371	0.82	2.05E−01	7.46E−01	1
	PSMC2	7	102 984 852	103 009 842	15	4	202 807	1.60	5.45E−02	6.78E−01	1	13	3	46 930	−1.70	9.55E−01	9.84E−01	1
	PSMB1	6	170 844 204	170 862 417	32	2	202 807	1.34	9.02E−02	7.25E−01	1	41	4	47 264	2.28	1.15E−02	3.59E−01	1
	PSMA2	7	42 956 460	42 971 805	18	1	202 807	1.23	1.09E−01	7.52E−01	1	23	2	47 477	0.56	2.89E−01	7.92E−01	1
	PSME2	14	24 612 574	24 615 855	1	1	202 807	1.02	1.53E−01	7.97E−01	1							
	SHFM1	7	96 318 079	96 339 203	32	3	202 807	0.95	1.72E−01	8.09E−01	1							
	PSMB11	14	23 511 376	23 513 269	2	1	202 807	0.94	1.74E−01	8.11E−01	1	1	1	45 652	−0.29	6.15E−01	9.12E−01	1
	PSMD13	11	236 808	252 984	63	5	202 807	0.85	1.99E−01	8.28E−01	1	63	6	47 258	0.49	3.12E−01	8.03E−01	1
	PSMA7	20	60 711 783	60 718 514	13	1	202 807	0.55	2.92E−01	8.81E−01	1	14	1	47 402	−0.37	6.43E−01	9.18E−01	1

Supplementary Table 2 **Detailed information about genes on four pathways annotated by MAGMA. *FEN1* is associated with colorectal cancer susceptibility both in East Asian and European populations. Genes on four pathways annotated by MAGMA in Biobank JAPAN but not in GECCO were left in blank** (continued)

Pathway	Gene	CHR	Start	Stop	Biobank Japan							GECCO						
					NSNPS	NPARAM	*N*	ZSTAT	*P*	*FDR*	Bonferroni	NSNPS	NPARAM	*N*	ZSTAT	*P*	*FDR*	Bonferroni
**Proteasome**	*POMP*	13	29 233 141	29 253 094	36	3	202 807	0.54	2.94E−01	8.83E−01	1	29	2	46 316	0.77	2.21E−01	7.55E−01	1
	PSMB4	1	151 372 041	151 374 412	3	1	202 807	0.52	3.01E−01	8.83E−01	1	2	1	47 429	0.74	2.29E−01	7.63E−01	1
	PSMD11	17	30 771 481	30 810 337	16	2	202 807	0.48	3.14E−01	8.91E−01	1	23	4	47 079	−1.53	9.37E−01	9.81E−01	1
	PSMD3	17	38 137 021	38 154 213	48	3	202 807	0.37	3.54E−01	9.10E−01	1	50	5	47 357	−0.76	7.76E−01	9.50E−01	1
	PSMD8	19	38 865 190	38 874 464	17	4	202 807	0.29	3.85E−01	9.18E−01	1	3	2	46 085	−0.60	7.26E−01	9.38E−01	1
	PSMF1	20	1 093 872	1 149 017	74	8	202 807	0.25	4.01E−01	9.19E−01	1	88	13	47 203	0.55	2.90E−01	7.92E−01	1
	PSMD1	2	231 921 578	232 037 541	126	10	202 807	0.17	4.34E−01	9.25E−01	1	97	6	47 230	−0.03	5.12E−01	8.85E−01	1
	PSMD14	2	162 164 786	162 268 228	87	6	202 807	0.16	4.35E−01	9.25E−01	1	114	10	47 338	−0.30	6.16E−01	9.13E−01	1
	PSMD2	3	184 016 886	184 026 842	6	2	202 807	0.15	4.41E−01	9.27E−01	1	2	1	45 496	0.11	4.56E−01	8.70E−01	1
	PSMA4	15	78 832 747	78 841 562	33	4	202 807	0.06	4.75E−01	9.33E−01	1	21	2	47 131	2.79	2.66E−03	1.98E−01	1
	PSMC3	11	47 440 320	47 448 024	14	2	202 807	0.06	4.76E−01	9.33E−01	1	11	3	47 223	1.75	3.99E−02	4.87E−01	1
	PSMC1	14	90 722 894	90 738 969	65	2	202 807	0.00	4.99E−01	9.40E−01	1	47	2	47 271	0.58	2.82E−01	7.92E−01	1
	PSMB2	1	36 040 259	36 107 445	27	3	202 807	−0.08	5.31E−01	9.47E−01	1	5	1	47 187	3.11	9.37E−04	1.26E−01	1
	PSMC6	14	53 173 894	53 194 716	17	3	202 807	−0.13	5.52E−01	9.50E−01	1	18	3	47 361	−1.00	8.42E−01	9.62E−01	1
	PSMD6	3	63 996 225	64 009 686	22	4	202 807	−0.22	5.86E−01	9.57E−01	1	18	3	46 935	1.86	3.17E−02	4.68E−01	1
	PSMD7	16	74 330 673	74 340 186	6	2	202 807	−0.26	6.02E−01	9.60E−01	1	7	3	47 288	−0.28	6.09E−01	9.11E−01	1
	PSMB5	14	23 485 752	23 504 429	5	1	202 807	−0.30	6.18E−01	9.65E−01	1	21	4	46 980	0.74	2.29E−01	7.63E−01	1
	PSMB8	6	32 808 494	32 812 712	11	4	202 807	−0.48	6.85E−01	9.70E−01	1	8	2	47 009	1.26	1.03E−01	6.37E−01	1
	PSMC4	19	40 476 912	40 487 671	16	1	202 807	−0.48	6.86E−01	9.70E−01	1	2	1	45 353	−1.50	9.34E−01	9.81E−01	1
	PSMA8	18	23 713 816	23 773 319	94	6	202 807	−0.55	7.10E−01	9.71E−01	1	34	3	47 056	−1.34	9.09E−01	9.74E−01	1
	PSME4	2	54 091 204	54 197 977	189	10	202 807	−0.65	7.42E−01	9.77E−01	1	197	12	47 135	−1.15	8.75E−01	9.68E−01	1
	PSMB9	6	32 821 927	32 827 628	18	3	202 807	−0.71	7.62E−01	9.80E−01	1	15	2	47 460	−0.11	5.44E−01	8.94E−01	1
	PSMA1	11	14 526 422	14 665 180	120	11	202 807	−0.98	8.37E−01	9.91E−01	1	105	12	47 286	1.03	1.52E−01	7.00E−01	1
	PSMB7	9	127 115 744	127 177 752	73	6	202 807	−1.13	8.71E−01	9.91E−01	1	40	2	47 040	2.51	6.08E−03	2.84E−01	1
	PSMC5	17	61 904 770	61 909 387	5	1	202 807	−1.39	9.17E−01	9.97E−01	1	5	2	46 206	0.64	2.62E−01	7.80E−01	1
	IFNG	12	68 548 550	68 553 521	4	2	202 807	−1.45	9.27E−01	9.99E−01	1	3	1	47 262	−1.20	8.85E−01	9.71E−01	1

Supplementary Table 2 **Detailed information about genes on four pathways annotated by MAGMA. *FEN1* is associated with colorectal cancer susceptibility both in East Asian and European populations. Genes on four pathways annotated by MAGMA in Biobank JAPAN but not in GECCO were left in blank** (continued)

Pathway	Gene	CHR	Start	Stop	Biobank Japan							GECCO						
					NSNPS	NPARAM	*N*	ZSTAT	*P*	*FDR*	Bonferroni	NSNPS	NPARAM	*N*	ZSTAT	*P*	*FDR*	Bonferroni
	PSMD12	17	65 336 619	65 362 721	37	5	202 807	−1.52	9.35E−01	1.00E+00	1	25	5	46 402	−1.22	8.88E−01	9.71E−01	1
**Proteasome**	*PSMA6*	14	35 747 811	35 786 680	11	3	202 807	−1.97	9.76E−01	1.00E+00	1	65	6	47 294	−0.03	5.13E−01	8.85E−01	1
	PSMB6	17	4 699 439	4 701 798	7	3	202 807	−2.15	9.84E−01	1.00E+00	1							
	POLR2H	3	184 079 502	184 086 383	11	2	202 807	1.90	2.87E−02	5.99E−01	1	2	1	45 733	1.34	9.04E−02	6.12E−01	1
	POLR2K	8	101 162 839	101 166 230	7	2	202 807	1.62	5.23E−02	6.76E−01	1	5	2	46 323	0.47	3.19E−01	8.06E−01	1
	POLR3K	16	96 979	103 632	9	1	202 807	1.56	5.91E−02	6.79E−01	1	5	1	46 286	0.06	4.76E−01	8.76E−01	1
	POLR1A	2	86 253 451	86 333 278	177	9	202 807	1.05	1.46E−01	7.92E−01	1	98	8	47 344	0.01	4.96E−01	8.84E−01	1
	POLR3GL	1	145 456 236	145 476 081	2	1	202 807	1.00	1.59E−01	8.01E−01	1	2	1	46 715	−0.64	7.38E−01	9.40E−01	1
	POLR3H	22	41 921 803	41 940 479	8	1	202 807	0.89	1.85E−01	8.20E−01	1	22	2	47 409	1.54	6.21E−02	5.54E−01	1
	ZNRD1	6	30 029 017	30 032 686	19	4	202 807	0.71	2.38E−01	8.50E−01	1							
	POLR3F	20	18 448 033	18 465 287	35	5	202 807	0.58	2.80E−01	8.74E−01	1	31	4	47 186	0.55	2.90E−01	7.92E−01	1
	POLR2D	2	128 603 840	128 615 729	28	4	202 807	0.47	3.18E−01	8.95E−01	1	30	5	46 678	1.12	1.32E−01	6.77E−01	1
	POLR1C	6	43 484 777	43 497 114	4	2	202 807	0.35	3.63E−01	9.13E−01	1	7	3	47 073	0.18	4.29E−01	8.62E−01	1
	POLR3G	5	89 769 876	89 810 474	66	7	202 807	0.33	3.72E−01	9.17E−01	1	65	9	47 102	−1.41	9.20E−01	9.77E−01	1
	POLR2C	16	57 496 551	57 505 921	9	2	202 807	0.33	3.72E−01	9.17E−01	1	13	4	47 290	0.11	4.55E−01	8.70E−01	1
	POLR1B	2	113 298 676	113 334 727	73	5	202 807	0.32	3.76E−01	9.17E−01	1	83	8	47 300	0.78	2.18E−01	7.55E−01	1
**RNA polymerase**	*POLR1E*	9	37 485 945	37 503 694	94	2	202 807	0.31	3.79E−01	9.17E−01	1	90	3	47 151	−0.70	7.59E−01	9.46E−01	1
	POLR3B	12	106 751 436	106 903 976	235	10	202 807	0.28	3.88E−01	9.19E−01	1	223	10	47 206	0.61	2.71E−01	7.85E−01	1
	POLR2F	22	38 349 674	38 368 463	18	2	202 807	0.20	4.20E−01	9.20E−01	1	16	2	47 142	−1.49	9.32E−01	9.81E−01	1
	POLR2J	7	102 113 547	102 119 446	14	2	202 807	−0.17	5.67E−01	9.54E−01	1	4	1	46 250	−1.14	8.74E−01	9.68E−01	1
	POLR2B	4	57 844 944	57 897 334	94	8	202 807	−0.20	5.79E−01	9.57E−01	1	122	13	47 412	1.51	6.61E−02	5.68E−01	1
	POLR2I	19	36 604 611	36 606 206	2	1	202 807	−0.44	6.71E−01	9.69E−01	1	3	1	46 198	−0.59	7.21E−01	9.38E−01	1
	POLR1D	13	28 194 880	28 241 559	88	12	202 807	−0.49	6.88E−01	9.70E−01	1	112	17	47 158	−0.25	5.98E−01	9.08E−01	1
	POLR3C	1	145 592 605	145 610 972	13	2	202 807	−0.51	6.95E−01	9.71E−01	1	14	2	47 012	0.37	3.55E−01	8.27E−01	1
	POLR2E	19	1 086 578	1 095 391	12	3	202 807	−0.53	7.03E−01	9.71E−01	1	11	3	46 655	−0.91	8.19E−01	9.58E−01	1
	POLR3A	10	79 734 907	79 789 298	57	4	202 807	−0.66	7.46E−01	9.77E−01	1	18	3	47 329	−1.00	8.40E−01	9.62E−01	1
	POLR2L	11	839 721	842 529	8	1	202 807	−0.71	7.61E−01	9.80E−01	1	11	2	46 962	0.86	1.95E−01	7.33E−01	1
	POLR2G	11	62 529 011	62 534 187	3	1	202 807	−1.28	8.99E−01	9.96E−01	1	1	1	47 242	−2.38	9.91E−01	9.96E−01	1
	POLR3D	8	22 102 619	22 108 680	7	2	202 807	−1.29	9.01E−01	9.96E−01	1	13	3	46 674	−1.03	8.49E−01	9.65E−01	1
	POLR2A	17	7 387 698	7 417 935	95	7	202 807	−2.66	9.96E−01	1.00E+00	1	87	4	47 162	1.34	8.99E−02	6.12E−01	1

Abbreviations: NSNPS, the number of SNPs annotated to that gene that were found in the data and were not excluded based on internal SNP QC; NPARAM, the number of relevant parameters used in the model; *N*, the sample size used when analyzing that gene; ZSTAT, the *Z*-value for the gene, based on its (permutation) *P*-value.

Supplementary Table 3 **Results for gene-set analysis at genomic level**

Pathways	Biobank Japan				GECCO			
	Beta	Beta_std	*SE*	*P*	Beta	Beta_std	*SE*	*P*
DNA replication	0.00	0.00	0.13	0.50	0.14	0.01	0.15	0.16
Mismatch repair	0.12	0.00	0.17	0.23	0.18	0.01	0.20	0.18
RNA polymerase	0.08	0.00	0.17	0.32	−0.16	−0.01	0.15	0.86
Proteasome	−0.22	−0.01	0.14	0.94	−0.08	0.00	0.14	0.70

Supplementary Table 4 **Eighty-six immune cells analyzed both in CIBERSORT and xCell algorithm**

Algorithm	Immune cells	Cell types	Group	Algorithm	Immune cells	Cell types	Group
	B cells naive	Adaptive immune cells	Lymphoids		$CD4^+$ central memory T-cells	$CD4^+$ Tcm	Lymphoids
	B cells memory	Adaptive immune cells	Lymphoids		$CD4^+$ effector memory T-cells	$CD4^+$ Tem	Lymphoids
	Plasma cells	Adaptive immune cells	Lymphoids		Memory B-cells	Memory B-cells	Lymphoids
	T cells CD8	Adaptive immune cells	Lymphoids		$CD8^+$ central memory T-cells	$CD8^+$ Tcm	Lymphoids
	T cells CD4 naive	Adaptive immune cells	Lymphoids		naive B-cells	naive B-cells	Lymphoids
	T cells CD4 memory resting	Adaptive immune cells	Lymphoids		$CD4^+$ memory T-cells	$CD4^+$ memory T-cells	Lymphoids
	T cells CD4 memory activated	Adaptive immune cells	Lymphoids		pro B-cells	pro B-cells	Lymphoids
	T cells follicular helper	Adaptive immune cells	Lymphoids		Class-switched memory B-cells	Class-switched memory B-cells	Lymphoids
	T cells regulatory (Tregs)	Adaptive immune cells	Lymphoids		Type 2 T-helper cells	Th2 cells	Lymphoids
	T cells gamma delta	Adaptive immune cells	Lymphoids		Type 1 T-helper cells	Th1 cells	Lymphoids
	NK cells resting	Innate immune cells	Lymphoids		$CD8^+$ naive T-cells	$CD8^+$ naive T-cells	Lymphoids
CIBERSORT	NK cells activated	Innate immune cells	Lymphoids		Natural killer T-cells	NKT	Lymphoids
	Monocytes	Innate immune cells	Myeloids		Gamma delta T-cells	Tgd cells	Lymphoids
	Macrophages M0	Innate immune cells	Myeloids		Endothelial cells	Endothelial cells	Stromal cells
	Macrophages M1	Innate immune cells	Myeloids		Smooth muscle cells	Smooth muscle cells	Stromal cells
	Macrophages M2	Innate immune cells	Myeloids		Fibroblasts	Fibroblasts	Stromal cells
	Dendritic cells resting	Innate immune cells	Myeloids		Chondrocytes	Chondrocytes	Stromal cells
	Dendritic cells activated	Innate immune cells	Myeloids		Adipocytes	Adipocytes	Stromal cells
	Mast cells resting	Innate immune cells	Myeloids		Microvascular endothelial cells	mv Endothelial cells	Stromal cells
	Mast cells activated	Innate immune cells	Myeloids		Myocytes	Myocytes	Stromal cells
	Eosinophils	Innate immune cells	Myeloids		Lymphatic endothelial cells	ly Endothelial cells	Stromal cells
	Neutrophils	Innate immune cells	Myeloids	xCell	Mesenchymal stem cells	MSC	Stromal cells
	Monocytes	Monocytes	Myeloids		Osteoblasts	Osteoblast	Stromal cells
	Macrophages	Macrophages	Myeloids		Preadipocytes	Preadipocytes	Stromal cells
	Dendritic cells	DC	Myeloids		Skeletal muscle cells	Skeletal muscle cells	Stromal cells
	Neutrophils	Neutrophils	Myeloids		Pericytes	Pericytes	Stromal cells
	Eosinophils	Eosinophils	Myeloids		Mesangial cells	Mesangial cells	Stromal cells
	Macrophages M1	Macrophages M1	Myeloids		Epithelial cells	Epithelial cells	Other
	Macrophages M2	Macrophages M2	Myeloids		Keratinocytes	Keratinocytes	Other
	Activated dendritic cells	aDC	Myeloids		Melanocytes	Melanocytes	Other
	Basophils	Basophils	Myeloids		Astrocytes	Astrocytes	Other
	Conventional dendritic cells	cDC	Myeloids		Neurons	Neurons	Other
xCell	Plasmacytoid dendritic cells	pDC	Myeloids		Hepatocytes	Hepatocytes	Other
	Immature dendritic cells	iDC	Myeloids		Sebocytes	Sebocytes	Other
	Mast cells	Mast cells	Myeloids		Erythrocytes	Erythrocytes	Stem cells
	$CD8^+$ T-cells	$CD8^+$ T-cells	Lymphoids		Multipotent progenitors	MPP	Stem cells
	NK cells	NK cells	Lymphoids		Common myeloid progenitors	CMP	Stem cells
	$CD4^+$ naive T-cells	$CD4^+$ naive T-cells	Lymphoids		Granulocyte-macrophage progenitors	GMP	Stem cells
	B-cells	B-cells	Lymphoids		Megakaryocyte–erythroid progenitors	MEP	Stem cells
	$CD4^+$ T-cells	$CD4^+$ T-cells	Lymphoids		Hematopoietic stem cells	HSC	Stem cells
	$CD8^+$ effector memory T-cells	$CD8^+$ Tem	Lymphoids		Megakaryocytes	Megakaryocytes	Stem cells
	Regulatory T-cells	Tregs	Lymphoids		Common lymphoid progenitors	CLP	Stem cells
	Plasma cells	Plasma cells	Lymphoids		Platelets	Platelets	Stem cells

Supplementary Table 5 **Major reagents used in multiplex immunofluorescence**

Name	Producer	Code	Dilution
Anti-CD3 primary antibody	Servicebio	GB13440	1 : 1000
Anti-CD45 primary antibody	Servicebio	GB113885	1 : 3000
Anti-CD22 primary antibody	Proteintech	66103-1-IG	1 : 3000
Anti-FEN1 primary antibody	Proteintech	14768-1-AP	1 : 200
HRP-conjugated goat anti-mouse secondary antibody	Servicebio	GB23301	1 : 500
HRP-conjugated goat anti-rabbit secondary antibody	Servicebio	GB23303	1 : 500
HRP-conjugated goat anti-mouse secondary antibody	Servicebio	GB23301	1 : 500
594-conjugated goat anti-rabbit fluorescent secondary antibody	Jackson		1 : 400
DAPI	Servicebio	G1012	
FITC-TSA	Servicebio	G1222	1 : 1000
CY3-TSA	Servicebio	G1223	1 : 2000
647-TSA	Servicebio	G1224	1 : 1000

Supplementary Table 6 **Intersection results of enriched pathways calculated from GSEA**

ID	Description	GSE74602				GSE106582				GSE117606				Nanjing colorectal cancer cohort				TCGA			
		Enrichment score	NES	*P* -value	*Q*-value	Enrichment score	NES	*P*- value	*Q*-value	Enrichment score	NES	*P*- value	*Q*-value	Enrichment score	NES	*P*- value	*Q*-value	Enrichment score	NES	*P*- value	*Q*-value
hsa03030	DNA replication	0.77	2.23	6.31E−07	8.38E−06	0.79	1.94	3.34E−06	5.92E−05	0.76	2.17	1.96E−07	3.07E−06	0.77	2.30	2.69E−08	6.27E−07	0.70	2.13	5.40E−06	6.75E−05
hsa03008	Ribosome biogenesis in eukaryotes	0.77	2.45	1.00E−10	3.51E−09	0.78	2.25	1.70E−10	4.19E−08	0.70	2.28	1.48E−09	6.03E−08	0.74	2.50	1.00E−10	5.24E−09	0.64	2.22	1.54E−08	6.93E−07
hsa03050	Proteasome	0.70	2.13	2.44E−06	2.70E−05	0.76	2.00	3.22E−06	5.92E−05	0.74	2.17	1.28E−07	2.37E−06	0.67	2.09	3.65E−06	4.25E−05	0.57	1.80	5.21E−04	2.86E−03
hsa03430	Mismatch repair	0.69	1.82	1.33E−03	4.37E−03	0.72	1.63	7.04E−03	3.21E−02	0.73	1.90	2.85E−04	1.35E−03	0.71	1.92	2.50E−04	1.47E−03	0.63	1.78	5.84E−03	1.80E−02
hsa03013	Nucleocytoplasmic transport	0.70	2.39	1.00E−10	3.51E−09	0.70	2.15	2.93E−09	3.61E−07	0.68	2.31	1.00E−10	7.77E−09	0.66	2.35	1.00E−10	5.24E−09	0.54	2.02	7.81E−07	1.35E−05
hsa03020	RNA polymerase	0.69	1.94	2.07E−04	9.16E−04	0.67	1.62	8.63E−03	3.69E−02	0.64	1.76	3.42E−03	8.79E−03	0.61	1.76	1.82E−03	6.79E−03	0.62	1.84	1.90E−03	8.41E−03
hsa03420	Nucleotide excision repair	0.67	2.04	1.47E−05	1.27E−04	0.67	1.73	1.55E−03	8.89E−03	0.64	1.89	1.52E−04	7.52E−04	0.64	1.99	2.21E−05	2.02E−04	0.54	1.72	1.60E−03	7.36E−03
hsa04110	Cell cycle	0.70	2.43	1.00E−10	3.51E−09	0.63	1.96	7.92E−07	2.58E−05	0.64	2.28	1.00E−10	7.77E−09	0.63	2.34	1.00E−10	5.24E−09	0.52	1.99	4.95E−07	9.33E−06
hsa04657	IL-17 signaling pathway	0.61	2.04	3.24E−07	4.61E−06	0.63	1.88	2.02E−05	2.93E−04	0.62	2.12	1.47E−08	4.96E−07	0.60	2.11	5.69E−08	1.08E−06	0.51	1.85	5.13E−05	4.82E−04
hsa03040	Spliceosome	0.66	2.25	1.06E−10	3.51E−09	0.62	1.92	1.06E−06	2.61E−05	0.53	1.90	2.56E−06	3.06E−05	0.61	2.25	1.00E−10	5.24E−09	0.53	1.98	4.97E−07	9.33E−06
hsa03010	Ribosome	0.76	2.68	1.00E−10	3.51E−09	0.60	1.90	1.19E−06	2.65E−05	0.64	2.12	4.83E−08	1.23E−06	0.56	2.08	1.53E−08	4.59E−07	0.56	2.17	8.06E−10	6.05E−08
hsa03018	RNA degradation	0.66	2.14	1.16E−07	2.31E−06	0.56	1.61	3.49E−03	1.91E−02	0.53	1.72	7.42E−04	2.79E−03	0.52	1.75	2.53E−04	1.47E−03	0.45	1.59	4.05E−03	1.38E−02
hsa04080	Neuroactive ligand-receptor interaction	−0.41	−1.69	9.89E−07	1.23E−05	−0.47	−1.53	1.35E−04	1.33E−03	−0.40	−1.58	1.42E−04	7.41E−04	−0.40	−1.58	5.57E−05	4.32E−04	−0.49	−1.88	1.00E−10	1.13E−08

ID	Description	GSE74602				GSE106582				GSE117606				Nanjing Colorectal Cancer Cohort				TCGA			
		enrichmentScore	NES	pvalue	qvalues	enrichmentScore	NES	pvalue	qvalues	enrichmentScore	NES	pvalue	qvalues	enrichmentScore	NES	pvalue	qvalues	enrichmentScore	NES	pvalue	qvalues
hsa04020	Calcium signaling pathway	−0.47	−1.90	7.12E−08	1.57E−06	−0.50	−1.57	1.62E−04	1.48E−03	−0.48	−1.79	7.79E−06	6.88E−05	−0.41	−1.55	4.38E−04	2.35E−03	−0.50	−1.84	1.17E−07	3.77E−06
hsa04921	Oxytocin signaling pathway	−0.43	−1.66	4.51E−04	1.79E−03	−0.50	−1.49	5.24E−03	2.75E−02	−0.53	−1.89	5.02E−06	5.10E−05	−0.43	−1.57	2.56E−03	8.38E−03	−0.46	−1.61	5.66E−04	3.04E−03
hsa04726	Serotonergic synapse	−0.43	−1.60	1.79E−03	5.42E−03	−0.52	−1.51	6.59E−03	3.15E−02	−0.55	−1.87	5.08E−05	3.56E−04	−0.43	−1.47	1.33E−02	2.90E−02	−0.51	−1.75	1.73E−04	1.30E−03
hsa04261	Adrenergic signaling in cardiomyocytes	−0.49	−1.86	8.65E−06	7.82E−05	−0.54	−1.61	5.45E−04	3.83E−03	−0.58	−2.06	6.05E−08	1.23E−06	−0.49	−1.74	6.42E−05	4.64E−04	−0.51	−1.81	5.27E−06	6.75E−05
hsa04022	cGMP-PKG signaling pathway	−0.51	−1.96	3.05E−07	4.61E−06	−0.56	−1.69	4.30E−05	5.30E−04	−0.58	−2.09	4.94E−08	1.23E−06	−0.52	−1.88	1.28E−06	1.68E−05	−0.55	−1.95	3.42E−08	1.28E−06
hsa04024	cAMP signaling pathway	−0.48	−1.93	1.88E−08	5.34E−07	−0.58	−1.81	9.42E−07	2.58E−05	−0.57	−2.13	1.15E−10	7.77E−09	−0.50	−1.89	1.63E−07	2.62E−06	−0.52	−1.93	7.05E−09	3.97E−07
hsa04270	Vascular smooth muscle contraction	−0.56	−2.10	4.24E−08	1.05E−06	−0.58	−1.70	2.01E−04	1.77E−03	−0.60	−2.10	1.41E−07	2.39E−06	−0.50	−1.76	8.73E−05	6.10E−04	−0.48	−1.65	3.97E−04	2.35E−03
hsa04928	Parathyroid hormone synthesis, secretion and action	−0.44	−1.62	1.89E−03	5.60E−03	−0.59	−1.68	4.93E−04	3.57E−03	−0.51	−1.75	3.88E−04	1.71E−03	−0.48	−1.64	1.23E−03	5.36E−03	−0.47	−1.58	3.25E−03	1.25E−02
hsa04713	Circadian entrainment	−0.47	−1.70	7.92E−04	2.92E−03	−0.59	−1.67	8.55E−04	5.54E−03	−0.53	−1.79	5.34E−04	2.26E−03	−0.44	−1.47	1.38E−02	2.98E−02	−0.54	−1.79	1.15E−04	9.66E−04
hsa04924	Renin secretion	−0.55	−1.88	1.59E−04	7.51E−04	−0.64	−1.73	5.93E−04	4.06E−03	−0.60	−1.88	1.11E−04	6.43E−04	−0.62	−1.94	1.12E−05	1.12E−04	−0.62	−1.98	8.05E−06	8.63E−05
hsa04971	Gastric acid secretion	−0.54	−1.83	1.30E−04	6.49E−04	−0.67	−1.84	8.27E−05	9.26E−04	−0.63	−2.00	6.25E−06	5.77E−05	−0.59	−1.90	4.94E−05	3.98E−04	−0.55	−1.80	2.14E−04	1.54E−03
hsa04976	Bile secretion	−0.55	−1.95	1.59E−05	1.32E−04	−0.68	−1.90	1.29E−05	1.99E−04	−0.68	−2.18	5.50E−08	1.23E−06	−0.68	−2.27	5.99E−10	2.51E−08	−0.59	−1.94	7.06E−06	7.96E−05
hsa04961	Endocrine and other factor-regulated calcium reabsorption	−0.54	−1.75	8.40E−04	3.04E−03	−0.68	−1.77	3.30E−04	2.54E−03	−0.62	−1.84	1.47E−04	7.49E−04	−0.56	−1.70	3.14E−03	9.82E−03	−0.58	−1.78	1.33E−03	6.53E−03
hsa04972	Pancreatic secretion	−0.51	−1.84	1.47E−04	7.15E−04	−0.70	−2.00	2.70E−07	1.33E−05	−0.70	−2.31	8.25E−10	4.19E−08	−0.63	−2.15	7.46E−08	1.30E−06	−0.57	−1.91	7.06E−06	7.96E−05
hsa00980	Metabolism of xenobiotics by cytochrome P450	−0.48	−1.63	2.84E−03	7.54E−03	−0.72	−1.98	9.25E−07	2.58E−05	−0.66	−1.94	7.12E−05	4.67E−04	−0.63	−1.99	5.05E−06	5.57E−05	−0.58	−1.89	2.90E−05	2.96E−04
hsa05204	Chemical carcinogenesis - DNA adducts	−0.51	−1.71	1.34E−03	4.37E−03	−0.72	−1.96	3.37E−06	5.92E−05	−0.68	−1.98	1.03E−04	6.35E−04	−0.69	−2.17	1.77E−08	4.63E−07	−0.65	−2.07	1.73E−06	2.43E−05
hsa00140	Steroid hormone biosynthesis	−0.52	−1.71	3.55E−03	8.61E−03	−0.75	−1.98	4.10E−06	6.73E−05	−0.68	−1.99	4.63E−05	3.36E−04	−0.58	−1.79	3.36E−04	1.85E−03	−0.58	−1.81	3.42E−04	2.08E−03
hsa00830	Retinol metabolism	−0.62	−2.08	4.63E−06	4.61E−05	−0.77	−2.08	1.63E−07	1.01E−05	−0.64	−1.88	3.32E−04	1.53E−03	−0.64	−2.02	2.52E−06	3.11E−05	−0.65	−2.07	1.73E−06	2.43E−05
hsa04978	Mineral absorption	−0.67	−2.24	2.37E−07	4.29E−06	−0.77	−2.03	4.74E−07	1.95E−05	−0.71	−2.12	1.01E−06	1.28E−05	−0.71	−2.19	5.49E−08	1.08E−06	−0.69	−2.14	1.98E−07	5.57E−06
hsa00071	Fatty acid degradation	−0.56	−1.73	2.97E−03	7.77E−03	−0.77	−1.93	1.08E−04	1.16E−03	−0.71	−2.04	4.62E−05	3.36E−04	−0.62	−1.80	9.45E−04	4.40E−03	−0.59	−1.74	2.34E−03	9.93E−03
hsa00982	Drug metabolism - cytochrome P450	−0.58	−1.97	3.75E−05	2.58E−04	−0.77	−2.09	2.87E−08	2.36E−06	−0.75	−2.18	7.05E−07	1.01E−05	−0.71	−2.22	2.43E−09	8.48E−08	−0.66	−2.12	2.98E−07	7.45E−06
hsa04960	Aldosterone-regulated sodium reabsorption	−0.67	−2.02	4.51E−05	2.80E−04	−0.78	−1.93	1.33E−04	1.33E−03	−0.74	−2.07	1.79E−05	1.40E−04	−0.77	−2.16	5.02E−07	7.01E−06	−0.60	−1.72	3.90E−03	1.37E−02
hsa00040	Pentose and glucuronate interconversions	−0.58	−1.68	6.58E−03	1.34E−02	−0.82	−1.96	2.39E−05	3.28E−04	−0.85	−2.07	9.83E−06	8.32E−05	−0.73	−2.02	1.60E−05	1.53E−04	−0.70	−1.93	2.78E−04	1.78E−03
hsa04964	Proximal tubule bicarbonate reclamation	−0.73	−1.99	1.85E−04	8.38E−04	−0.83	−1.85	3.06E−04	2.43E−03	−0.85	−2.18	7.48E−07	1.01E−05	−0.82	−2.09	9.23E−06	9.67E−05	−0.70	−1.81	1.51E−03	7.24E−03
hsa00910	Nitrogen metabolism	−0.81	−2.05	6.14E−05	3.59E−04	−0.85	−1.80	6.13E−04	4.08E−03	−0.87	−2.08	4.10E−06	4.38E−05	−0.81	−1.92	2.12E−04	1.35E−03	−0.72	−1.77	4.20E−03	1.39E−02

Supplementary Table 7 **Pathways enriched from GSEA and GSVA at protein level**

ID	Description	Enrichment score	NES	*P* value	Q-values
hsa03008	Ribosome biogenesis in eukaryotes	0.74	2.69	1.00E−10	2.08E−09
hsa03030	***DNA replication***	0.71	2.22	4.30E−06	3.50E−05
hsa03410	***Base excision repair***	0.69	1.91	5.15E−04	1.68E−03
hsa00100	***Steroid biosynthesis***	0.67	1.71	7.52E−03	1.36E−02
hsa03430	***Mismatch repair***	0.65	1.79	4.28E−03	8.90E−03
hsa04110	***Cell cycle***	0.65	2.36	1.71E−09	2.77E−08
hsa03013	Nucleocytoplasmic transport	0.63	2.44	1.00E−10	2.08E−09
hsa03420	***Nucleotide excision repair***	0.61	1.94	4.44E−04	1.50E−03
hsa03020	***RNA polymerase***	0.60	1.69	6.52E−03	1.19E−02
hsa03040	***Spliceosome***	0.59	2.38	1.00E−10	2.08E−09
hsa04657	IL-17 signaling pathway	0.56	1.91	1.74E−04	7.59E−04
hsa05219	Bladder cancer	0.56	1.63	1.43E−02	2.30E−02
hsa04115	p53 signaling pathway	0.55	1.77	2.56E−03	5.88E−03
hsa03050	***Proteasome***	0.55	1.82	9.96E−04	2.66E−03
hsa03015	mRNA surveillance pathway	0.54	1.99	1.13E−05	8.34E−05
hsa00970	***Aminoacyl-tRNA biosynthesis***	0.51	1.71	4.11E−03	8.66E−03
hsa03010	***Ribosome***	0.50	2.02	6.39E−07	5.72E−06
hsa04120	Ubiquitin mediated proteolysis	0.49	1.86	8.44E−05	4.44E−04
hsa05230	Central carbon metabolism in cancer	0.47	1.63	1.24E−02	2.06E−02
hsa03018	***RNA degradation***	0.45	1.59	1.04E−02	1.80E−02
hsa05202	Transcriptional misregulation in cancer	0.40	1.48	1.42E−02	2.30E−02
hsa04144	Endocytosis	−0.35	−1.37	1.74E−02	2.69E−02
hsa04151	PI3K-Akt signaling pathway	−0.38	−1.45	1.37E−02	2.24E−02
hsa05163	Human cytomegalovirus infection	−0.40	−1.46	1.23E−02	2.06E−02
hsa05014	Amyotrophic lateral sclerosis	−0.40	−1.60	3.94E−04	1.36E−03
hsa05207	Chemical carcinogenesis - receptor activation	−0.43	−1.50	1.69E−02	2.63E−02
hsa04360	***Axon guidance***	−0.45	−1.56	6.36E−03	1.17E−02
hsa04062	Chemokine signaling pathway	−0.45	−1.59	4.85E−03	9.87E−03
hsa05205	Proteoglycans in cancer	−0.45	−1.64	9.20E−04	2.58E−03
hsa05022	Pathways of neurodegeneration - multiple diseases	−0.47	−1.91	5.19E−09	7.74E−08
hsa04022	cGMP-PKG signaling pathway	−0.48	−1.59	8.80E−03	1.54E−02
hsa04921	Oxytocin signaling pathway	−0.48	−1.59	8.52E−03	1.51E−02
hsa04510	Focal adhesion	−0.48	−1.76	1.23E−04	5.92E−04
hsa05010	***Alzheimer disease***	−0.48	−1.89	7.46E−08	8.90E−07
hsa04020	***Calcium signaling pathway***	−0.48	−1.60	5.41E−03	1.04E−02
hsa04142	Lysosome	−0.49	−1.68	8.87E−04	2.56E−03
hsa04014	Ras signaling pathway	−0.49	−1.73	4.72E−04	1.56E−03
hsa04261	Adrenergic signaling in cardiomyocytes	−0.50	−1.63	5.39E−03	1.04E−02
hsa04936	Alcoholic liver disease	−0.50	−1.69	1.44E−03	3.62E−03
hsa04612	Antigen processing and presentation	−0.50	−1.57	1.49E−02	2.36E−02
hsa05322	Systemic lupus erythematosus	−0.51	−1.58	9.73E−03	1.69E−02
hsa05100	Bacterial invasion of epithelial cells	−0.51	−1.66	3.95E−03	8.51E−03
hsa05016	***Huntington disease***	−0.51	−1.97	6.83E−09	9.41E−08
hsa04728	Dopaminergic synapse	−0.51	−1.69	2.94E−03	6.57E−03
hsa05145	Toxoplasmosis	−0.51	−1.70	1.60E−03	3.91E−03
hsa04926	Relaxin signaling pathway	−0.52	−1.71	2.44E−03	5.67E−03
hsa04918	Thyroid hormone synthesis	−0.53	−1.60	1.51E−02	2.36E−02
hsa05410	***Hypertrophic cardiomyopathy***	−0.54	−1.63	1.24E−02	2.06E−02
hsa04730	***Long-term depression***	−0.55	−1.64	7.92E−03	1.42E−02
hsa05012	***Parkinson disease***	−0.55	−2.12	1.05E−10	2.08E−09
hsa04270	***Vascular smooth muscle contraction***	−0.55	−1.80	6.21E−04	1.89E−03
hsa05020	***Prion disease***	−0.56	−2.15	1.00E−10	2.08E−09
hsa04371	Apelin signaling pathway	−0.56	−1.87	1.03E−04	5.27E−04
hsa04024	cAMP signaling pathway	−0.56	−1.90	7.22E−05	3.98E−04
hsa04640	***Hematopoietic cell lineage***	−0.56	−1.72	4.05E−03	8.63E−03
hsa04724	Glutamatergic synapse	−0.57	−1.76	1.65E−03	3.94E−03
hsa05414	***Dilated cardiomyopathy***	−0.57	−1.72	4.98E−03	1.00E−02

Supplementary Table 7 **Pathways enriched from GSEA and GSVA at protein level**(continued)

ID	Description	Enrichment score	NES	*P* value	Q-values
hsa05034	Alcoholism	−0.57	−1.88	1.39E−04	6.23E−04
hsa05146	Amoebiasis	−0.58	−1.90	2.58E−04	1.05E−03
hsa03320	***PPAR signaling pathway***	−0.58	−1.82	7.44E−04	2.18E−03
hsa04974	Protein digestion and absorption	−0.58	−1.85	2.81E−04	1.11E−03
hsa05412	***Arrhythmogenic right ventricular cardiomyopathy***	−0.58	−1.76	2.85E−03	6.46E−03
hsa00640	***Propanoate metabolism***	−0.59	−1.63	1.84E−02	2.82E−02
hsa05415	Diabetic cardiomyopathy	−0.59	−2.20	1.35E−10	2.41E−09
hsa04714	Thermogenesis	−0.60	−2.24	1.00E−10	2.08E−09
hsa05208	Chemical carcinogenesis - reactive oxygen species	−0.60	−2.28	1.00E−10	2.08E−09
hsa05416	Viral myocarditis	−0.60	−1.84	6.24E−04	1.89E−03
hsa00565	Ether lipid metabolism	−0.60	−1.62	1.48E−02	2.36E−02
hsa04727	GABAergic synapse	−0.60	−1.72	5.55E−03	1.05E−02
hsa00620	***Pyruvate metabolism***	−0.60	−1.81	9.91E−04	2.66E−03
hsa04932	Non-alcoholic fatty liver disease	−0.60	−2.15	1.67E−08	2.13E−07
hsa04512	***ECM-receptor interaction***	−0.61	−1.92	1.26E−04	5.92E−04
hsa04514	***Cell adhesion molecules***	−0.62	−2.06	5.81E−07	5.48E−06
hsa04923	Regulation of lipolysis in adipocytes	−0.62	−1.69	6.21E−03	1.16E−02
hsa04610	Complement and coagulation cascades	−0.62	−2.03	2.48E−05	1.58E−04
hsa04725	Cholinergic synapse	−0.63	−1.95	7.34E−05	3.98E−04
hsa04925	Aldosterone synthesis and secretion	−0.63	−1.88	3.93E−04	1.36E−03
hsa00860	Porphyrin metabolism	−0.63	−1.75	1.09E−02	1.85E−02
hsa04976	Bile secretion	−0.63	−1.86	1.05E−03	2.76E−03
hsa04924	Renin secretion	−0.64	−1.85	9.47E−04	2.61E−03
hsa04726	Serotonergic synapse	−0.64	−1.96	4.48E−05	2.77E−04
hsa04060	***Cytokine-cytokine receptor interaction***	−0.64	−1.72	5.37E−03	1.04E−02
hsa05143	African trypanosomiasis	−0.64	−1.72	5.11E−03	1.02E−02
hsa04971	Gastric acid secretion	−0.64	−1.90	3.81E−04	1.36E−03
hsa04916	***Melanogenesis***	−0.65	−1.92	2.86E−04	1.11E−03
hsa04940	***Type I diabetes mellitus***	−0.66	−1.75	3.33E−03	7.26E−03
hsa04260	***Cardiac muscle contraction***	−0.66	−1.99	1.12E−04	5.57E−04
hsa04972	Pancreatic secretion	−0.66	−2.12	1.79E−06	1.53E−05
hsa00020	***Citrate cycle (TCA cycle)***	−0.66	−1.85	1.16E−03	2.97E−03
hsa04713	Circadian entrainment	−0.67	−2.01	4.94E−05	2.90E−04
hsa05032	Morphine addiction	−0.67	−1.92	3.68E−04	1.36E−03
hsa04961	Endocrine and other factor-regulated calcium reabsorption	−0.68	−1.98	1.88E−04	8.00E−04
hsa00140	Steroid hormone biosynthesis	−0.69	−1.78	3.30E−03	7.26E−03
hsa00190	***Oxidative phosphorylation***	−0.69	−2.44	1.00E−10	2.08E−09
hsa04080	***Neuroactive ligand-receptor interaction***	−0.69	−1.81	1.54E−03	3.82E−03
hsa00380	***Tryptophan metabolism***	−0.72	−1.98	3.95E−04	1.36E−03
hsa04672	***Intestinal immune network for IgA production***	−0.72	−1.87	1.08E−03	2.81E−03
hsa05332	***Graft-versus-host disease***	−0.73	−1.86	1.62E−03	3.93E−03
hsa00830	***Retinol metabolism***	−0.73	−1.99	1.36E−04	6.23E−04
hsa00280	Valine, leucine and isoleucine degradation	−0.73	−2.23	5.82E−07	5.48E−06
hsa05204	Chemical carcinogenesis - DNA adducts	−0.73	−2.07	2.38E−05	1.58E−04
hsa04723	Retrograde endocannabinoid signaling	−0.74	−2.48	1.00E−10	2.08E−09
hsa05320	***Autoimmune thyroid disease***	−0.74	−1.91	5.55E−04	1.74E−03
hsa05330	***Allograft rejection***	−0.74	−1.91	5.55E−04	1.74E−03
hsa00511	***Other glycan degradation***	−0.74	−1.77	4.51E−03	9.28E−03
hsa00071	Fatty acid degradation	−0.75	−2.14	6.30E−06	4.90E−05
hsa00410	***beta-Alanine metabolism***	−0.75	−1.92	7.15E−04	2.13E−03
hsa04975	Fat digestion and absorption	−0.76	−2.04	5.03E−05	2.90E−04
hsa00980	***Metabolism of xenobiotics by cytochrome P450***	−0.77	−2.26	8.35E−08	9.34E−07
hsa04964	***Proximal tubule bicarbonate reclamation***	−0.78	−1.94	3.51E−04	1.34E−03
hsa00910	Nitrogen metabolism	−0.78	−1.82	2.43E−03	5.67E−03
hsa00650	***Butanoate metabolism***	−0.79	−1.97	2.11E−04	8.78E−04
hsa00920	Sulfur metabolism	−0.79	−1.74	5.53E−03	1.05E−02
hsa00982	***Drug metabolism - cytochrome P450***	−0.80	−2.29	1.30E−07	1.37E−06
hsa00053	***Ascorbate and aldarate metabolism***	−0.80	−1.90	9.24E−04	2.58E−03
hsa00350	***Tyrosine metabolism***	−0.81	−2.10	1.17E−05	8.34E−05
hsa00040	***Pentose and glucuronate interconversions***	−0.82	−2.07	2.05E−05	1.41E−04

Bold italic fonts indicate the 51 shared pathways in GSEA and GSVA results. Abbreviation: NES, normalized enrichment score.

Supplementary Table 8 **Functional annotation of rs4246215 by FAVOR**

Category	Functional elements	Score
Basic	Variant	chr11-61796827-G-T
Basic	rsID	rs4246215
Basic	TOPMed QC Status	PASS
Basic	TOPMed Depth	39.45
Variant category	Gencode Comprehensive Category	UTR3
Variant category	Gencode Comprehensive Info	FEN1 (ENST00000305885.3:c.*323G>T)
Variant category	UCSC Info	ENST00000305885.2 (ENST00000305885.2:c.*323G>T)
Variant category	Disruptive Missense	NO
Variant category	RefSeq Info	FEN1 (NM_004111:c.*323G>T)
Variant category	CAGE Promoter	NO
Variant category	CAGE Enhancer	NO
Variant category	GeneHancer	NO
Variant category	SuperEnhancer	NO
Clinvar	Clinical Significance	NA
Chromatin states	cHmm E1	1
Chromatin states	cHmm E6	5
Chromatin states	cHmm E7	15
Chromatin states	cHmm E8	8
Chromatin states	cHmm E9	4
Chromatin states	cHmm E10	4
Chromatin states	cHmm E11	1
Chromatin states	cHmm E15	10
Conservation	priPhCons	0.73
Conservation	mamPhCons	0
Conservation	verPhCons	0
Conservation	priPhyloP	0.6
Conservation	mamPhyloP	1.76
Conservation	verPhyloP	1.61
Conservation	GerpN	5.16
Conservation	GerpS	0.55
Epigenetics	DNase	0.37
Epigenetics	H2AFZ	4.56
Epigenetics	H3K27ac	7.05
Epigenetics	H3K4me1	8.73
Epigenetics	H3K4me2	15.1
Epigenetics	H3K4me3	7.17
Epigenetics	H3K9ac	6.25
Epigenetics	H4K20me1	10.56
Epigenetics	H3K9me3	3.3
Epigenetics	H3K27me3	2.78
Epigenetics	H3K36me3	21.66
Epigenetics	H3K79me2	27.19
Epigenetics	totalRNA	26.84
Epigenetics	GC	0.456953642
Epigenetics	CpG	0
Intergrative score	LINSIGHT	0.06
Intergrative score	FATHMM-XF noncoding	0.03
Intergrative score	CADD RawScore	0.929445
Intergrative score	CADD PHRED	12.34

Supplementary Table 8 **Functional annotation of rs4246215 by FAVOR** (continued)

Category	Functional elements	Score
Intergrative score	aPC-Epigenetics	6.93
Intergrative score	aPC-Conservation	11.61
Intergrative score	aPC-Protein-Function	2.97
Intergrative score	aPC-Local-Nucleotide-Diversity	8.27216255
Intergrative score	aPC-Mutation-Density	15.64589212
Intergrative score	aPC-Transcription-Factor	1.18
Intergrative score	aPC-Mappability	0.33
Intergrative score	aPC-Proximity-To-TSS-TES	14.82
Intergrative score	Funseq Description	noncoding
Local nucleotide diversity	RecombinationRate	0
Local nucleotide diversity	NuclearDiversity	1.55
Local nucleotide diversity	bStatistic	794
Mappability	Umap k100	1
Mappability	Bismap k100	0.52
Mappability	Umap k50	0.7
Mappability	Umap k24	0.38
Mutation density	Common100bp	1
Mutation density	Rare100bp	0
Mutation density	Sngl100bp	12
Mutation density	Common1000bp	1
Mutation density	Rare1000bp	3
Mutation density	Sngl1000bp	122
Mutation density	Common10000bp	5
Mutation density	Rare10000bp	34
Mutation density	Sngl10000bp	1291
Proximity table	minDistTSS	887
Proximity table	minDistTSE	406

Abbreviation: FAVOR: Functional Annotation of Variants-Online Resource (http://favor.genohub.org/).

Supplementary Table 9 **Detailed information about genetic effect of rs4246215 on immunity (TCGA)**

Algorithm	SNP	Immune cells	Beta	*P*
CIBERSORT	rs4246215	Macrophages M0	1.43E−02	8.36E−02
		Dendritic cells activated	−1.75E−03	9.95E−02
		Mast cells activated	4.05E−03	1.25E−01
		T cells follicular helper	−3.32E−03	2.24E−01
		B cells naive	−3.54E−03	2.42E−01
		Macrophages M2	−5.18E−03	2.44E−01
		Monocytes	−1.05E−03	2.46E−01
		T cells CD4 memory activated	−1.62E−03	2.58E−01
		T cells gamma delta	2.21E−05	3.14E−01
		NK cells activated	7.72E−04	3.52E−01
		Neutrophils	−1.33E−03	4.00E−01
		T cells CD8	2.49E−03	4.61E−01
		Dendritic cells resting	3.50E−04	4.87E−01
		Plasma cells	−2.37E−03	5.22E−01
		T cells CD4 naive	−2.37E−04	6.82E−01
		T cells CD4 memory resting	−1.01E−03	8.39E−01
		Eosinophils	−3.65E−05	9.01E−01
		Macrophages M1	−3.57E−04	9.05E−01

Supplementary Table 9 **Detailed information about genetic effect of rs4246215 on immunity (TCGA)** (continued)

Algorithm	SNP	Immune cells	Beta	*P*
CIBERSORT	rs4246215	Mast cells resting	−1.24E−04	9.32E−01
		NK cells resting	−7.46E−05	9.68E−01
		B cells memory	−6.84E−06	9.90E−01
		T cells regulatory Tregs	1.59E−05	9.94E−01
xCell	rs4246215	**Basophils**	5.40E−03	**6.65E−03**
		**Eosinophils**	1.47E−03	**1.46E−02**
		**CD4 Tem**	1.45E−03	**3.07E−02**
		**Mesangial cells**	1.44E−03	**4.28E−02**
		**Th2 cells**	−7.92E−03	**4.30E−02**
		MSC	1.26E−02	5.12E−02
		aDC	1.04E−02	6.91E−02
		DC	1.08E−03	7.29E−02
		Megakaryocytes	−4.08E−04	7.81E−02
		Macrophages M2	8.22E−04	9.45E−02
		Macrophages M1	1.87E−03	1.05E−01
		CD4 Tcm	1.25E−03	1.09E−01
		Plasma cells	8.78E−04	1.15E−01
		Macrophages	2.02E−03	1.26E−01
		Adipocytes	1.73E−03	1.38E−01
		CD8 Tcm	1.78E−03	1.44E−01
		NK cells	1.23E−04	1.68E−01
		Pericytes	4.78E−03	1.88E−01
		cDC	2.31E−03	2.14E−01
		Class switched memory B cells	1.06E−03	2.47E−01
		CLP	−3.76E−03	2.56E−01
		iDC	8.02E−03	2.80E−01
		HSC	−4.36E−03	2.99E−01
		Hepatocytes	3.54E−05	3.15E−01
		B cells	2.38E−03	3.25E−01
		Smooth muscle	−7.83E−03	3.59E−01
		Skeletal muscle	4.18E−05	3.70E−01
		Endothelial cells	−2.29E−03	3.82E−01
		Erythrocytes	3.32E−06	4.25E−01
		Platelets	7.83E−05	5.41E−01
		CD8 naive T cells	−2.44E−04	5.68E−01
		pDC	−6.59E−04	5.75E−01
		Keratinocytes	−3.74E−04	6.29E−01
		naive B cells	2.22E−04	6.32E−01
		NKT	−1.42E−03	6.36E−01
		Sebocytes	1.63E−04	6.49E−01
		Myocytes	−1.35E−04	6.52E−01
		Melanocytes	4.83E−05	6.58E−01
		Neurons	6.54E−05	6.72E−01
		CD8 Tem	8.63E−05	6.87E−01
		pro B cells	−1.71E−04	6.88E−01
		Preadipocytes	1.95E−04	6.99E−01
		CD4 naive T cells	2.57E−04	7.05E−01
		Th1 cells	1.53E−03	7.06E−01
		Mast cells	−1.73E−04	7.40E−01
		ly Endothelial cells	−4.42E−04	7.41E−01
		Neutrophils	−1.23E−04	7.54E−01
		Osteoblast	1.52E−04	7.61E−01
		Chondrocytes	−4.55E−04	7.73E−01
		Tgd cells	−2.47E−05	7.76E−01
		GMP	1.03E−04	7.82E−01
		Epithelial cells	7.82E−04	7.94E−01
		Fibroblasts	−6.27E−04	7.96E−01
		Monocytes	−2.46E−04	7.97E−01
		CD8 T cells	2.10E−04	8.21E−01
		mv Endothelial cells	3.01E−04	8.22E−01
		CD4 T cells	−1.89E−05	8.69E−01
		Memory B cells	8.01E−05	8.92E−01
		Tregs	−4.05E−05	9.15E−01
		MEP	1.37E−04	9.36E−01
		Astrocytes	3.33E−04	9.40E−01
		CD4 memory T cells	3.84E−05	9.70E−01
		CMP	0.00E+00	1.00E+00
		MPP	0.00E+00	1.00E+00

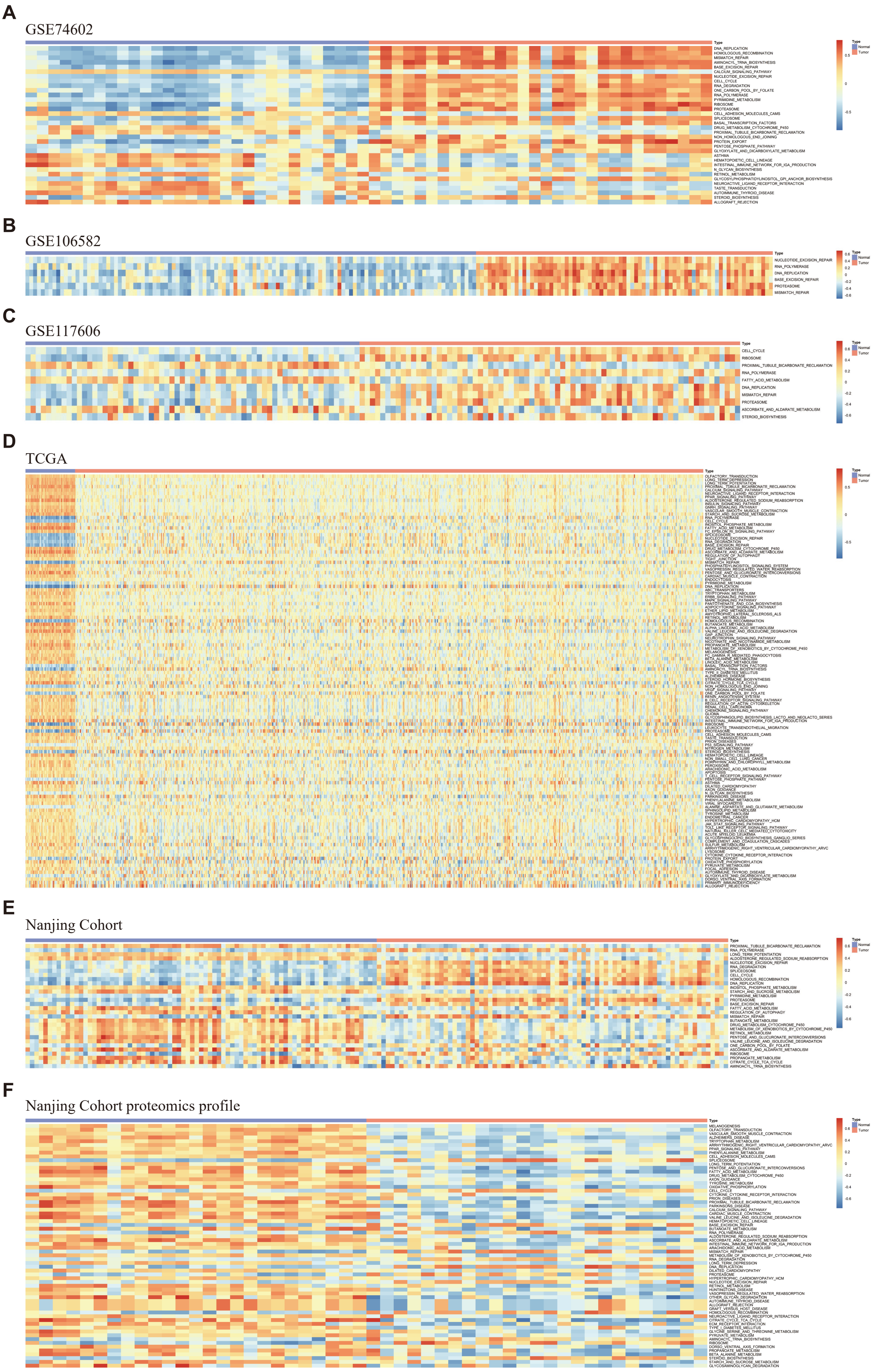

Supplementary Fig. 1 **Activity estimation of pathways identified by GSVA at the RNA and protein levels.** GSE74602 (A), GSE106582 (B), GSE117606 (C), TCGA (D), Nanjing cohort (E), and Nanjing cohort proteomics profiles (F). Abbreviations: GSVA, gene set variation analysis; TCGA, The Cancer Genome Atlas.

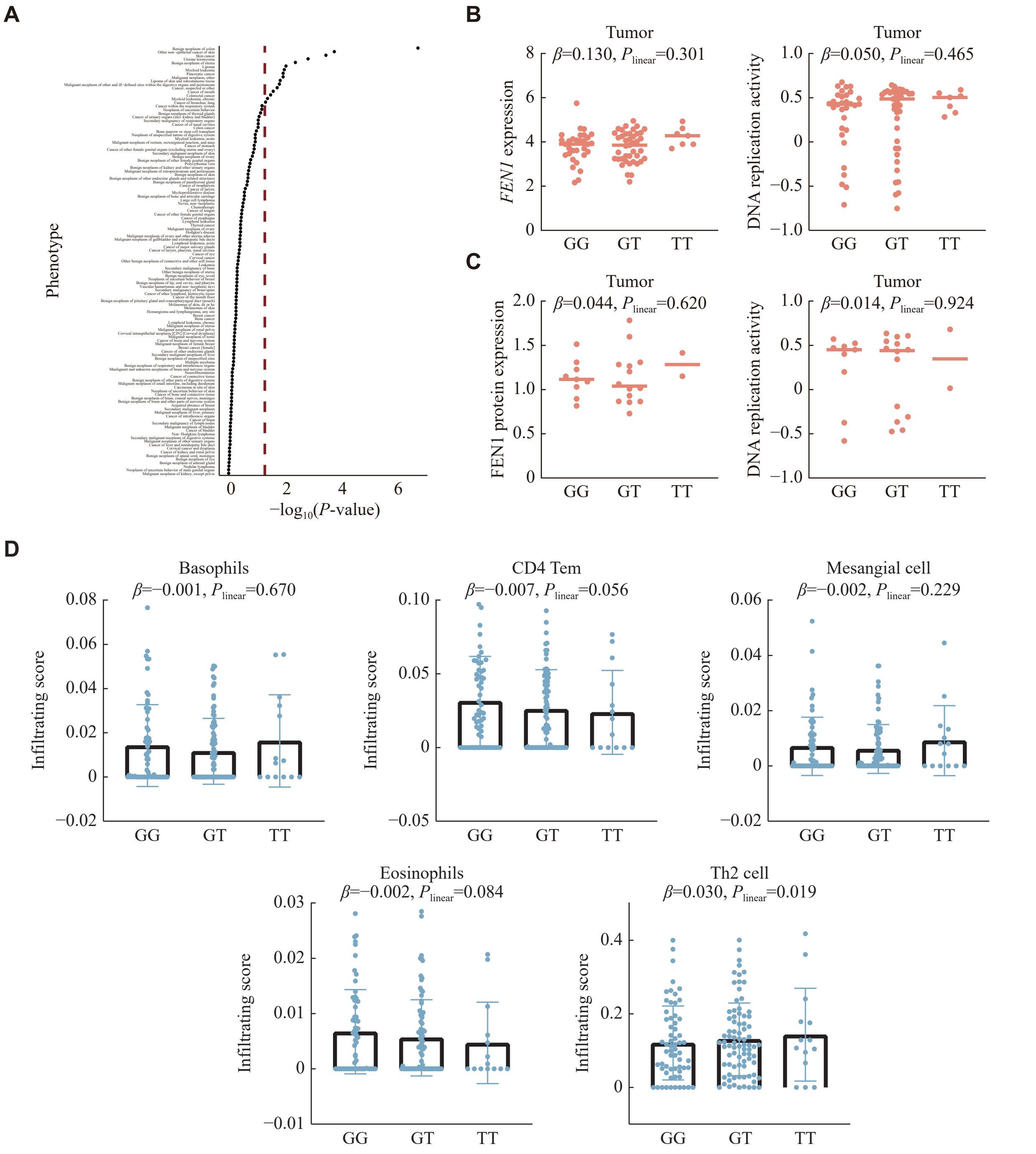

Supplementary Fig. 2 **Genetic effect of rs4246215.** A: Genetic associations between rs4246215 and multiple traits. The vertical dashed red line represents a *P*-value < 0.05. B: Genetic effect of rs4246215 on *FEN1* and DNA replication at the RNA level (Nanjing cohort). C: Genetic effect of rs4246215 on FEN1 and DNA replication at the protein level (Nanjing cohort). D: Genetic effect of rs4246215 on immunity (Nanjing cohort). Abbreviation: Tem, effector memory T cells.

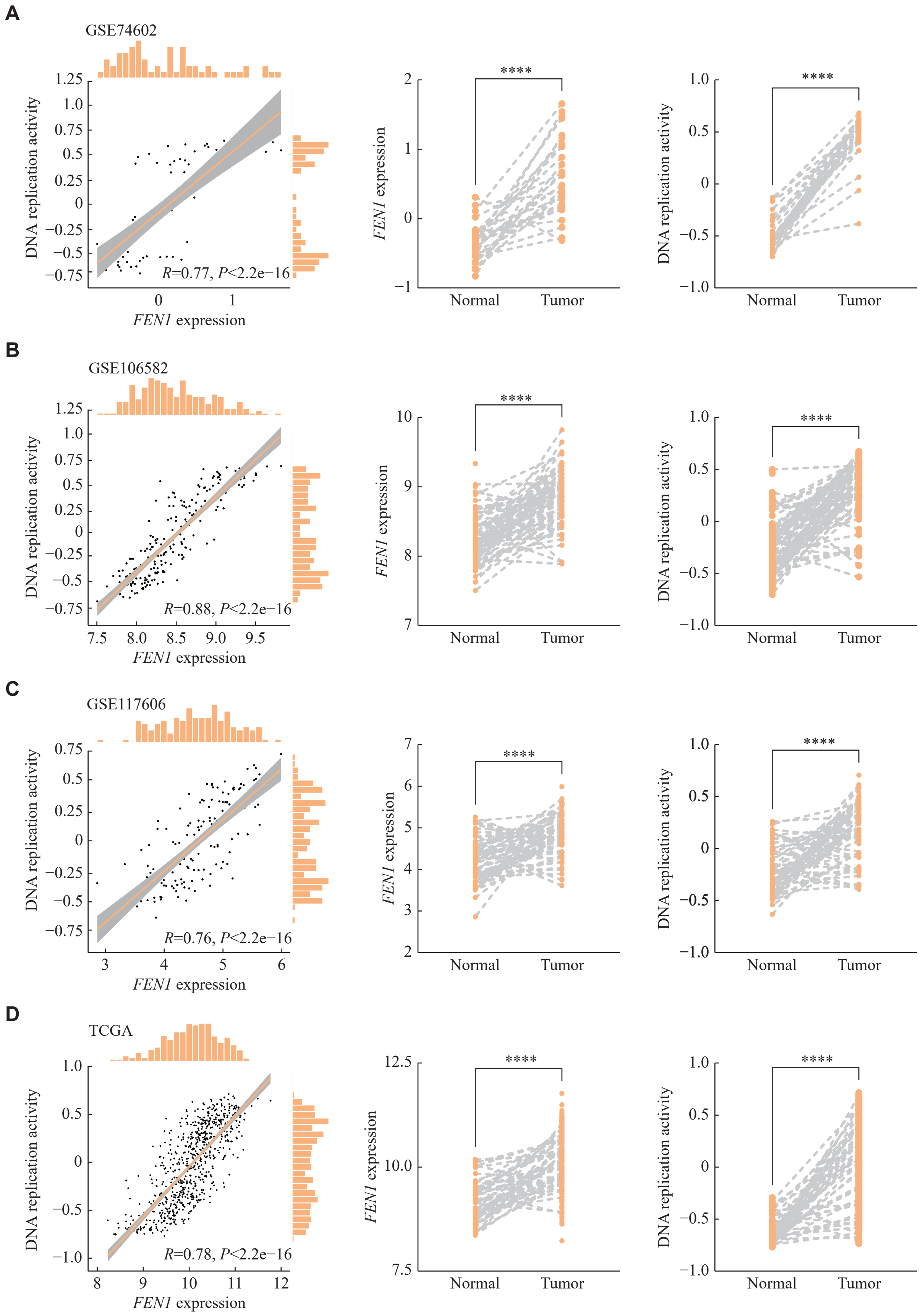

Supplementary Fig. 3 **Expression pattern of *FEN1* and DNA replication activity and their correlation at the RNA level.** GSE74602 (A), GSE106582 (B), GSE117606 (C), TCGA (D). Abbreviation: TCGA, The Cancer Genome Atlas.*****P* < 0.000 1.

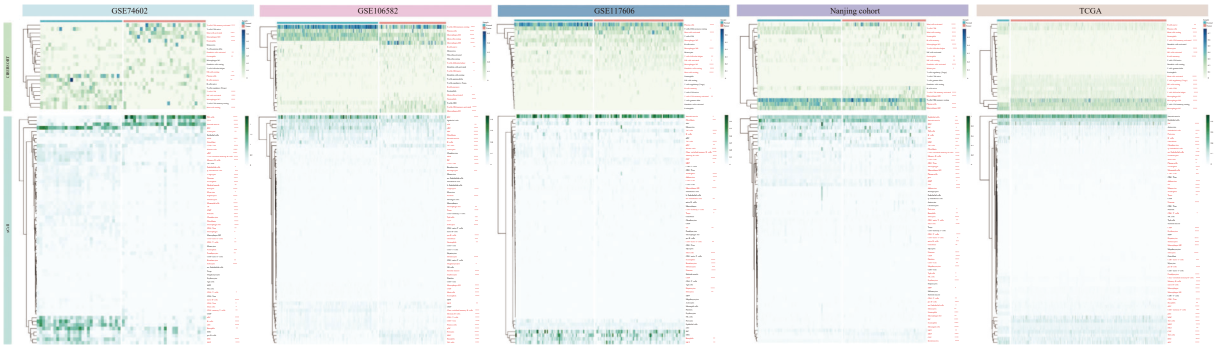

Supplementary Fig. 4 **Landscape of immune and stromal cell infiltration in colorectal tumors and normal tissues.** The significant difference between two groups was compared by the Wilcoxon test. $^{*}P < 0.05$, $^{**}P < 0.01$, $^{***}P < 0.001$, and $^{****}P < 0.000\,1$.
